# Neuronal Deletion of Caspase 8 Protects against Brain Injury in Mouse Models of Controlled Cortical Impact and Kainic Acid-Induced Excitotoxicity

**DOI:** 10.1371/journal.pone.0024341

**Published:** 2011-09-16

**Authors:** Maryla Krajewska, Zerong You, Juan Rong, Christina Kress, Xianshu Huang, Jinsheng Yang, Tiffany Kyoda, Ricardo Leyva, Steven Banares, Yue Hu, Chia-Hung Sze, Michael J. Whalen, Leonardo Salmena, Razqallah Hakem, Brian P. Head, John C. Reed, Stan Krajewski

**Affiliations:** 1 Sanford-Burnham Medical Research Institute, La Jolla, California, United States of America; 2 Neuroscience Center, Massachusetts General Hospital, Harvard Medical School, Charlestown, Massachusetts, United States of America; 3 Department of Anesthesiology, University of California San Diego, La Jolla, California, United States of America; 4 VA San Diego Healthcare System, San Diego, California, United States of America; 5 Department of Medical Biophysics, Ontario Cancer Institute, Toronto, Ontario, Canada; Federal University of Rio de Janeiro, Brazil

## Abstract

**Background:**

Acute brain injury is an important health problem. Given the critical position of caspase 8 at the crossroads of cell death pathways, we generated a new viable mouse line (N*casp8* ^−/−^), in which the gene encoding caspase 8 was selectively deleted in neurons by *cre-lox* system.

**Methodology/Principal Findings:**

Caspase 8 deletion reduced rates of neuronal cell death in primary neuronal cultures and in whole brain organotypic coronal slice cultures prepared from 4 and 8 month old mice and cultivated up to 14 days *in vitro*. Treatments of cultures with recombinant murine TNFα (100 ng/ml) or TRAIL (250 ng/mL) plus cyclohexamide significantly protected neurons against cell death induced by these apoptosis-inducing ligands. A protective role of caspase 8 deletion *in vivo* was also demonstrated using a controlled cortical impact (CCI) model of traumatic brain injury (TBI) and seizure-induced brain injury caused by kainic acid (KA). Morphometric analyses were performed using digital imaging in conjunction with image analysis algorithms. By employing virtual images of hundreds of brain sections, we were able to perform quantitative morphometry of histological and immunohistochemical staining data in an unbiased manner. In the TBI model, homozygous deletion of caspase 8 resulted in reduced lesion volumes, improved post-injury motor performance, superior learning and memory retention, decreased apoptosis, diminished proteolytic processing of caspases and caspase substrates, and less neuronal degeneration, compared to wild type, homozygous *cre*, and caspase 8-floxed control mice. In the KA model, N*casp8* ^−/−^ mice demonstrated superior survival, reduced seizure severity, less apoptosis, and reduced caspase 3 processing. Uninjured aged knockout mice showed improved learning and memory, implicating a possible role for caspase 8 in cognitive decline with aging.

**Conclusions:**

Neuron-specific deletion of caspase 8 reduces brain damage and improves post-traumatic functional outcomes, suggesting an important role for this caspase in pathophysiology of acute brain trauma.

## Introduction

The loss of neurons underlies the pathophysiology of numerous neurological diseases. Understanding the key mediators of the neuronal cell death is critical to identification of potential targets for therapeutic intervention [1]. Caspases are highly conserved aspartate-specific cysteine proteases that have both apoptotic and non-apoptotic functions (reviewed in [2]).

Caspase 8 is the apical protease in the extrinsic apoptotic pathway activated at the plasma membrane by various TNF-family death receptors [3]. Stimulation of these receptors by pro-apoptotic ligands causes receptor clustering followed by the recruitment of FADD (Fas-associated death domain protein), which in turn binds Death Effector Domains (DEDs) in the proform of caspase 8, a protease that then cleaves and activates downstream effector caspases responsible for apoptosis [4]. In some types of cells, caspase 8 indirectly activates downstream caspases by cleaving the BH3-only protein Bid [5], leading to activation of the Apaf-1/caspase 9 apoptosome [6]. In some scenarios, caspase 8 activation can also occur downstream of the mitochondrial apoptotic pathway [7]. Moreover, an alternative pathway involving caspase 8 activation is associated with endoplasmic reticulum (ER) stress [8], [9], [10], [11]. Thus, caspase 8 bridges several death mechanisms, including extrinsic and intrinsic apoptosis pathways, ER stress, and possibly autophagic pathways (reviewed in [12]) [13].

A role for caspase 8 in neuronal cell death has been suggested by evidence of proteolytic processing and increases in caspase 8 protease activity in the settings of ischemia [14], [15] and seizures [16], [17]. However, attempts to genetically determine the function of caspase 8 in the brain have been hampered by the observation that *caspase 8* gene knockout in mice is embryonic lethal [18]. As an alternative, transgenic mice have been created that over-express the endogenous caspase 8 inhibitor, c-FLIP, indirectly suggesting that caspase 8 is a critical mediator of glucose deprivation–induced neuronal cell death [19]. However, c-FLIP has additional functions besides suppression of caspase 8, including induction of NF-κB family transcription factors (reviewed in [20]), making interpretation of these results difficult. To date, ablation of caspase 8 expression in the brain of living mice has not been described.

Given the critical position of caspase 8 at the crossroads of cell death pathways, we sought to determine the contribution of this caspase to neuronal injury *in vivo* by the generation and characterization of a new viable mouse line (N*casp8* ^−/−^), in which caspase 8 has been selectively deleted in neurons. Our findings demonstrate a protective role of caspase 8 deletion *in vivo* using a controlled cortical impact (CCI) model of traumatic brain injury (TBI) and seizure-induced brain injury caused by kainic acid (KA), as well as *in vitro* employing cultures of embryonal primary cortical neurons and adult brain coronal slices.

## Materials and Methods

Experimental protocols involving vertebrate animals were approved by the Institutional Animal Care and Use Committee (#07-043, 07-049, 10-087, and 10-089) of the Sanford-Burnham Medical Research Institute and comply with the National Institutes of Health (NIH) Guide for the Care and Use of Laboratory Animals.

### Generation of mouse line with neuronal ablation of caspase 8

The generation and characterization of the CRE3 transgenic line were summarized elsewhere [21], [22]. T-cell-lineage-specific *casp8* mutant mice with deletion of exons 3 and 4 of *caspase 8* (*casp8^fl3-4/fl3-4^; LckCre* mice) [23] were backcrossed for nine generations onto the FVB/N background to generate *casp8* ^fl/fl^ mice without *cre*. For deletion of the *caspase 8 (casp8)* gene specifically in neurons, homozygous *casp8*-floxed mice (*casp8^fl/fl^*) were bred with pan-neuronal CRE3 homozygous transgenic mice (FVB/N background) to obtain heterozygous founders (*casp8^fl/+^cre*). To generate homozygous congenic *casp8* ^fl/fl^CRE3 mice, the heterozygous founders were intercrossed for nine generations; the *Casp8*/CRE3 homozygous line (henceforth named N*casp8* ^−/−^) was subsequently used in most experimental procedures. Genotyping was undertaken by PCR using primers detecting *cre* and *casp8 ^flox^* alleles. A *cre* 750-bp band was detected by forward and reverse primers (5′-GCCTGCATTACCGGTCGATGCAACGA-3′ and 5′ GTGGCAGATGGCGCGGCAACACCATT-3′, respectively); 800-, 650- and 200-bp bands for unrecombined floxed *casp8* alleles, wild-type (WT) *casp8* mouse gene, as well as recombined floxed *casp8*, respectively, were obtained using forward and reverse primers ( *5′- GGC CTT CCT GAG TAC TGT CAC CTG T-3′* and *5′-CCA GGA AAA GAT TTG TGT CTA GC-3′* ).

### Primary neuronal culture (PNC), treatment and viability assay

Primary cortical neurons were derived from embryos (E16.5) of CRE3 and N*casp8* ^−/−^ homozygous mice and cultured as previously described [24]. Briefly, cortices were dissected under a microscope in ice-cold Mg^2+^ and Ca^2+^ -free Hank's Balanced Salt Solution (HBSS, Invitrogen, Carlsbad, CA, USA), and trypsinized (0.25% trypsin in HBSS) at 37°C for 15 min. The tissue was mildly triturated 5–10 times and filtered through a 40 µm cell strainer (BD Biosciences, San Diego, CA, USA). The isolated cells were counted by Trypan exclusion in a Neubauer chamber, then seeded at a density of 75,000 cells/cm^2^ on poly-D-lysine (Sigma-Aldrich, St. Louis, MO, USA) -coated cover slips in 24 well plates and cultured in Neurobasal medium supplemented with B27 (Invitrogen). After 3 or 14 days in culture, neurons were treated with recombinant murine TNFα (100 ng/ml; PeproTech, Rocky Hill, NJ, USA) and cycloheximide (CHX) (1 µg/ml; MP Biomedicals, Solon, OH, USA) for 24 h or staurosporine (1 µM for 4 h and 50 nM) overnight. To determine the proportion of viable neurons, cells were fixed, then stained with Hoechst dye to visualize nuclei and immunostained with an antibody to neuron-specific nuclear protein (NeuN) (Chemicon, Ramona, CA, USA; MAB377) followed by application of Alexa Fluor 594 conjugated anti-mouse antibody (Invitrogen). The ratio of neurons with NeuN immunoreactivity that showed Hoechst staining profile characteristic of viable cells to the total cell number (number of Hoechst-positive nuclei) [25], [26] was determined by counting cells on ten random fluorescent images generated by microscopy using an ×20 objective. In addition, to define the proportion of apoptotic/degenerating neurons in the 14-day-old culture, a polarity sensitive indicator for viability and apoptosis (pSIVA) (CytoGLO™ pSIVA-IANBD Apoptosis/Viability Microscopy Set Imgenex IMG-6701K), dialyzed into PBS, was applied to the PNC at a concentration of 10 µg/1 ml TC medium. In this test, fluorescently tagged annexin binds to externalized phosphatidylserine exposed on apoptotic cell membranes allowing for monitoring changes that occur at different stages of apoptosis in living cells [27]. After 15 min incubation, the cells were fixed and immunostained using MAP2 antibody. The ratio of pSIVA-positive neurons with colocalized MAP2 immunopositive reactions to total number of MAP2 positive cells was determined after incubation with Alexa Fluor 594 conjugated anti-mouse antibody, as described above. Using EVOS fluorescence microscope (Advanced Microscopy Group, Bothell, WA), 15 random digital images of cells were taken at 20× magnification from 3 wells per experiment for manual cell counting.

### Brain organotypic coronal slice cultures (BOCSC)

Brain slices were prepared and cultivated using a simple method for organotypic cultures of nervous tissue [28], slightly modified. Briefly, 4 and 8 month old mice, representing all three homozygous lines (3 mice/line), were anesthetized, and transcardial perfusion-flush was performed with PBS containing protease inhibitors (Sigma). After 2–3 minutes of perfusion, the mice were quickly decapitated, the brains promptly removed, and submerged in pre-cooled and 95% oxygenated ACSF (artificial cerebrospinal buffer). Subsequently, both hemispheres were separated from cerebellum and the brain stem, mounted on the vibrotome stage with a supportive agarose block to obtain ∼500–600 µm thick coronal sections of both hemispheres using a Leica VT1200S vibrotome. The slices were plated on organotypic inserts (Millicell 30 mm Organotypic cm PTFE 0.4 µm; Millipore/Chemicon) and maintained for 72 h in 6-well plates on the surface of Neurobasal A medium supplemented with B27 and GlutaMax under standard incubation conditions (5% CO_2_, 95% air). Representative slices at bregma levels from −1.22 to 3.08 were maintained in culture for 72 hours, and then treated with TRAIL (Calbiochem, La Jolla, CA, USA) at 250 ng/mL for 4 h. Following treatment, the slices were fixed in Z-fix solution and processed into paraffin blocks. Additional brain slices derived from CRE3 and N*casp8* ^−/−^ lines (6 mice/line) were cultured for 14 days for further processing. Histological and immunocytochemical results were generated using 5 µm thick serial paraffin sections numbered from 5 to 50, counting from the bottom aspect that faces the mesh and culture medium. The surface layer was removed by trimming before serial sections were collected. The sections were stained either with H&E or Masson's trichrome or subjected to TUNEL assay or immunostaining using antibodies specific for cleaved caspase 3 (Cell Signaling Technology, Danvers, MA, USA; cat.#9661 and Imgenex, San Diego, CA, USA; cat.#5699), MAP2 (clone HM-2), NeuN (clone A60 Millipore Chemicon, cat.# MAB377), β3-tubulin (Abcam; ab18207), GFAP (Abcam, Cambridge, UK; ab7260), S100 (DakoCytomation, Carpinteria, CA, USA; cat.# Z0311), and Iba1 (Wako Chemicals USA, Richmond, VA; Code No. 019-19741).

### Controlled Cortical Impact (CCI) model of Traumatic Brain Injury

Mice (12 to 28 weeks of age; weight 24–38 g) of wild-type, CRE3, *casp8^fl/fl^*, and *casp8^fl/fl^/cre* genotypes were anesthetized with 4% isoflurane (Aerrane; Baxter, UK) in 70% N_2_O and 30% O_2_ and positioned in a stereotaxic frame. Using a head restraint, a 5-mm craniotomy was made using a portable drill and a trephine over the right parietotemporal cortex and the bone flap was removed [29]. Mice were subjected to CCI using the benchmark stereotaxic impactor (Impact One™; myNeuroLab.com) with the actuator part mounted directly on the stereotaxic instrument. The impactor 3 mm tip accelerated down to the 1.0 mm distance, reaching the preset velocity of 3 m/s, and the applied electromagnetic force remained there for the dwell time of 85 ms, and then retracted automatically. The contact sensor indicated the exact point of contact for reproducible results. Face mask anesthesia (1–2% isoflurane in 70%/30% nitrous oxide/oxygen) was used during the entire procedure. Afterwards, the scalp was closed with sutures, anesthesia discontinued, and mice recovered in their cages. Sham operated mice underwent the same surgical procedures (craniotomy) with the exception of the traumatic impact. Naïve animals were not exposed to any surgical procedures. For the first 2 h post-CCI, mice were closely monitored in their cages. Histopathological and functional outcomes were compared between the control and knockout mice at 2 h, 6 h, 24 h, 48 h, and 3 weeks after the trauma (8–12 mice per time per experimental group). Sham animals were tested at 0 h and 21 days using 4–6 mice per group.

### Spatial learning and working memory performance

The Morris water maze (MWM) task was used to evaluate spatial memory performance as described previously [30], [31]. The test was performed in a water tank for mice (122 cm diameter, 75 cm deep; San Diego Instruments, Inc., USA) filled with water (24±1°C) to 29 cm depth and divided into four quadrants of equal size, with several well visible cues located on the walls of each quadrant. The first cued learning tests were performed using a visible black platform and a visible sign; performance was evaluated by measuring latency to find the platform. Each mouse was given four trials to find the platform. The purpose of this test was to assess whether the experimental groups differed in the motor or proximal visual skills necessary to locate and escape to the platform. In the next 3 days (the acquisition learning phase), mice learnt how to locate a clear plexiglass platform (5 cm in diameter) submerged 0.5 cm below the surface of the water in a quadrant different from that used in the cue trial (using external visual cues); the water was rendered opaque by white, non-toxic paint. The goal of this task was to evaluate how fast mice learn to escape from the water onto the platform after being randomly placed into the pool at one of four sites. The learning task was performed for 3 consecutive days for each mouse before surgery and consisted of 8 trials per day (morning and afternoon sessions) for a total of 24 trials. Each day the start quadrant was changed after the first trial starting from one of the points farthest from the platform. Once the mouse found the platform, it remained there for 10 s before beginning the next trial. The latency to reach and climb the hidden platform within 60 s was recorded. Mice that failed to reach the platform by 60 s, were placed on the platform and allowed to remain there for 10 s. Mice rested in a warming chamber for 4 min inter-trial intervals. Memory retention was tested before the brain trauma (the next day after 3-day training) and 1 and 3 weeks after CCI. All animals were subjected to a 1-min probe trial of free swimming in the maze without escape platform, and escape latency was scored. Data collection was automated using the Any-Maze Video Tracking System (San Diego Instruments, Inc.), thereby eliminating observer bias. A separate cohort of 18 month old mice (*casp8^fl/fl^* (n = 19) and N*casp8* ^−/−^ (n = 20)) with no brain injury was also subjected to the MWM procedure. Learning capability and extinction of memory were tested 40 (P2) and 60 (P3) days after the cued learning phase (P1).

### Motor function assessment by the wire grip test

Vestibulomotor function was assessed using a wire-grip test [31], [32]. Mice (n = 8–12/group) were placed on a metal wire (45 cm long) suspended 45 cm above a foam mat between 2 vertical bars. Mice were allowed to cross the wire for 60 s. The latency that a mouse remained on the wire within a 60 s interval was measured, and wire grip scores were quantified using a 5-point scale [31], [33]. Mice that were unable to remain on the wire for less than 30 s were given a score of zero. A score of one point was given if the mouse failed to hold on to the wire with both sets of fore paws and hind paws together; two points were given if the mice held on to the wire with both fore paws and hind paws but not the tail; three points were given if the mouse used its tail along with both fore paws and both hind paws; four points were given if the mouse moved along the wire on all four paws plus tail; and five points were given if mice that scored four points also ambulated down one of the posts used to support the wire. The wire grip test was performed in triplicate and an average value calculated for each mouse on each day of testing. For the statistical analysis, cumulative scores were produced from all three trials. The test was performed before CCI and repeated weekly after CCI through the entire duration of the experiment (21 days). An additional cohort of aged mice with no brain injury (described above) was also subjected to this test.

### Sensorimotor function evaluation using beam walking test

Beam walking is a motor skill and balance test. Prior to CCI, each mouse was trained to run across a 29.5 cm long×1.2 cm wide square wooden beam placed 33 cm above a table to reach its home cage located at the far end of the beam. Training was followed by a repeat test (probe 1) performed at one week before the CCI procedure, which was repeated 7 days (probe 2) and 21 days (probe 3) after the procedure. Performance was measured as the number of foot slips while traversing the length of the beam three times. Progressive impairment correlates with increased numbers of foot slips during crossing. The test was also performed on the separate cohort of aged uninjured mice (described above).

### Hindlimb flexion for testing paresis and asymmetry

The hindlimb flexion was elicited by gently lifting the animal by its tail and holding it 0.5 meters above a table [34]. The absence of asymmetry/paresis was scored as zero (no neurological abnormalities – “no”). The presence of asymmetry/paresis was scored as one (“yes”). The test was performed 1 week before CCI (probe 1) and 7 and 21 days after the procedure (probes 2 and 3, respectively).

### Tissue preparation and staining

Mice were anesthetized with Avertin (BD Franklin Lakes, NY, USA) and transcardially perfused with phosphate-buffered saline (PBS) pH 7.4, followed by zinc-buffered formalin (Z-fix; Anatech, Inc., Battle Creek, MI, USA). After decapitation, the whole head was fixed in the Z-fix solution. Following skull removal and 3–5 days of brain post-fixation, each brain was cut in 1.5 mm thick coronal slices fitting in one cassette to be processed and embedded into a single paraffin block. Thus, all sections from each coronal slice were stained on the same slide ensuring identical staining conditions. In addition to H&E, brain sections were stained with Masson's trichrome (American Master*Tech Scientific, Inc.; Lodi, CA, USA). Dewaxed tissue sections (4.0–5.0 µm) were immunostained as reported previously [35] using an antibody to mouse IgG (DakoCytomation), rabbit polyclonal antibodies to GFAP (Abcam), Iba1 (Wako Chemicals USA), phospho-Tau (Thr231) (Thermo Fisher Scientific, Lafayette, CO, USA; cat.#OPA1-03156), phospho-Tau (T181) (Abcam ab38505), cleaved caspase 3 (Cell Signaling Technology and Imgenex), and phospho-c-Jun (Cell Signaling Technology, cat.#9164), as well as mouse monoclonal antibodies to NeuN (clone A60; Millipore Chemicon), and microtubule-associated protein (MAP2) (clone HM-2; Sigma-Aldrich), and rat monoclonal antibody to Ly-6G (BD Pharmingen; cat#551459). Application of the primary antibody was followed by incubation with goat anti-mouse or goat anti-rabbit polymer-based EnVision-HRP-enzyme conjugate (DakoCytomation). Diaminobenzidine (DAB; DakoCytomation) and SG-Vector (Vector Lab, Inc.; Burlingame, CA, USA) chromogens were applied, yielding brown and black colors, respectively.

### Terminal deoxyribonucleotidyl transferase–mediated dUTP nick end-labeling (TUNEL) assay

The detection of nuclei with fragmented DNA by TUNEL was accomplished using the ApopTag Peroxidase *in situ* Apoptosis Detection Kit (Chemicon). Nuclear Red or hematoxylin were used as counterstains.

### Assessment of brain lesion volumes

We assessed volume of lesion and lesion cavity. Lesion volume was calculated as previously reported in detail by our group [35]. Briefly, Masson's trichrome–stained sections were digitalized using the Aperio scanning system (Aperio Technologies; Vista, CA, USA). Using the pen tool, virtual slides were annotated by encircling the lesion edges (penumbra) between intact and pathologically changed brain tissue, and the lesion area (mm^2^) was reported in the annotation window. The rostral–caudal dimensions of the foci were determined through the use of a stereotaxic atlas for the mouse brain [36], which was viewed electronically side by side with the coronal section images. Distance (mm) between coronal coordinates for bregma +1.54 (rostral position) and −3.88 (caudal position) determined the distribution of the lesion volume. Lesion volume was calculated by summing each lesion area multiplied by the distance between each coronal slice. The proposed calculation is based on Cavalieri's method [37] modified for the purpose of volumetric analysis performed on digital slides. The volumes of injury-induced cavities were determined separately in a similar manner.

### Quantitative analysis of immunostaining and histochemical staining

Quantitative analysis was performed as described previously [35]. Briefly, all slides were scanned at an absolute magnification of 400× [resolution of 0.25 µm/pixel (100,000 pix/in.)] using the Aperio ScanScope CS system (Aperio Technologies). The acquired digital images representing whole tissue sections were analyzed applying the Spectrum Analysis algorithm package and ImageScope analysis software (version 9; Aperio Technologies) to quantify IHC and histochemical stainings. These algorithms make use of a color deconvolution method [38] to separate stains. Algorithm parameters were set to achieve concordance with manual scoring on a number of high-power fields, including intensity thresholds for positivity and parameters that control cell segmentation using the nuclear algorithm. Colorimetric intensity measurements were applied to Masson's trichrome histochemical stain to obtain quantitative characteristics of brain tissue damage, as well as percentage and total number of degenerating neurons in the annotated area of interest [35]. In addition, NeuN and MAP2 immunohistochemistry were performed for assessment of neuronal cell preservation. Automated morphometric analyses were accomplished for entire regions of interest and not limited to a few selected fields.

### Neuropathological scoring of brain injury

Brain injury was evaluated by an observer masked to the study groups using a neuropathological scoring system: grade 0 - no observable injury; grade 1 - restricted cortical impact area with small petechial bleedings and variable degree of acute ischemic neuronal degeneration but no cortical tissue loss; grade 2 - necrotic lesion core and variable tissue loss of the injured cortex with penumbra demonstrating neuronal degeneration limited to cortex only; grade 3 - sporadic larger hematomas and ischemic, necrotic, spongiotic changes crossing the corpus callosum and involving ipsilateral hippocampal sectors CA1–CA3; and grade 4 - confluent impact core encompassing large cortical area, entire hippocampus and deeper thalamic structures in the upper quadrant of the ipsilateral hemisphere. The score was given to each brain coronal section containing a pathological focus, and the total score was a sum of all scores for the slices recorded for each animal.

### Immunoblotting

Immunoblotting methods were used to analyze proteolytic processing of caspases and their substrates in the brains of control and knockout mice after CCI [39]. Extracts from cortex and basal ganglia ipsi- and contralateral to the injury site, derived from N*casp8* ^−/−^, control CRE3 and *casp8^fl/fl^*, (n = 4–5 per group) were prepared, and 30 µg of protein per lane was analyzed by SDS-PAGE/immunobloting, probing blots with rabbit polyclonal antibodies to caspase 8 (Imgenex IMG**#**5703, #5704; Cell Signaling cat#4927), and caspase 3 (Imgenex #IMG-5699), as well as mouse monoclonal antibody to cleaved PARP (Asp214; 19F4; Cell Signaling #9546S). Antibody to β-actin (Sigma-Aldrich) served as a loading control. Detection was accomplished using an enhanced chemiluminescence (Amersham Biosciences, Piscataway, NJ, USA) multiple antigen detection immunoblotting method, as described previously [39].

### Induction of status epilepticus

Mice were injected intraperitoneally (i.p.) with kainic acid (KA) solution (K-0250, monohydrate, Sigma) at 25 or 35 mg/kg (n = 8–11 per group). Eight control and 11 N*casp8* ^−/−^ mice were used in this study. Animals were monitored during status epilepticus to determine duration and severity of seizure activity and the neurological status in post-seizure period.

### Seizure scoring

KA-induced seizures were scored every 15 min for 2 h according to a described procedure [40] with minor modifications [41]. Seizure activity was scored as follows: 1 - arrest of motion; 2 - myoclonic jerks of the head and neck with brief twitching movements; 3 - unilateral clonic activity; 4 - bilateral forelimb tonic and clonic activity; 5 – generalized tonic-clonic activity with loss of postural tone; 6 - death due to continuous convulsions.

### Statistical Analysis

Data were analyzed using the STATISTICA software package (StatSoft; Tulsa, OK, USA). Statistical analyses of pairs of control *vs* knockout mice or specimens were performed using a one-way Student's t-test. A one-way analysis of variance (ANOVA) method was used to determine the significance of differences of multiple point data sets. Box and whisker or column mean±SEM plots were applied to visualize the data in a graphic form. Mean values are plotted as a middle point at each time assessed after brain trauma or KA treatment, while the whiskers reflect ± standard error of mean (SEM). Chi-square test was performed to test whether the frequencies of neurological deficits, observed in the hindlimb flexion test, differed significantly from the expected ones. *P* values<0.05 were reported as statistically significant.

## Results

### Generation and characterization of mice with neuron-specific ablation of caspase 8 expression

**Figure 1:**
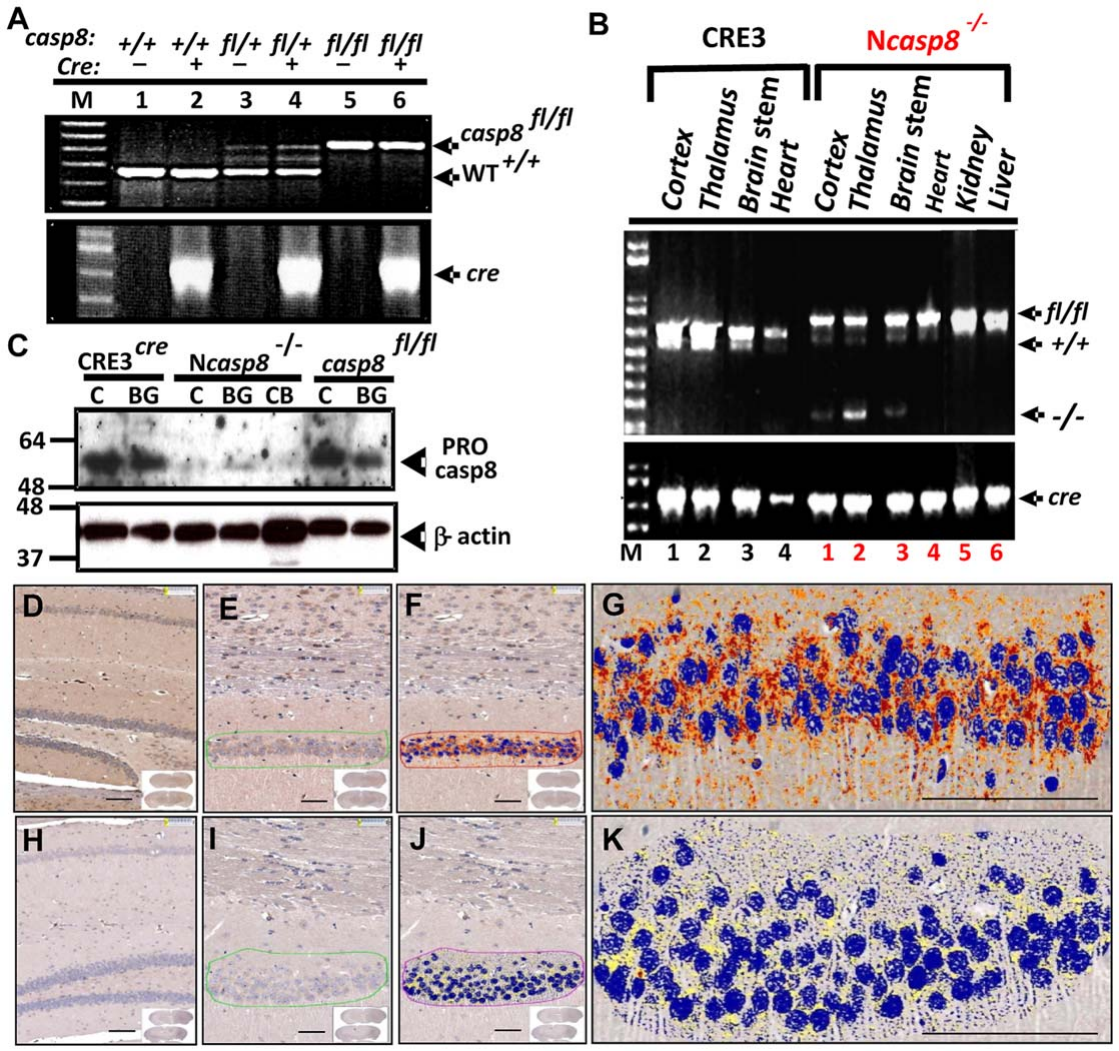
Generation of mouse line (Ncasp8^−/−^) with neuron-specific ablation of caspase8. (**A**) PCR was performed using 1 µg tail genomic DNA for transgenic CRE3 and caspase 8*^flox^* animals. PCR products were generated using amplification primers specific to either the *cre* or *casp8* genes. PCR products were analyzed by agarose gel-electrophoresis and ethidium bromide staining. Arrows indicate the *cre* PCR product of 750 bp and *casp8* PCR products of 800 bp for floxed genes and 650 bp for wild-type *casp8*. (**B**) Brain-specific recombination of floxed *casp8* genes in CRE3 mice. Genomic DNA was isolated from various tissues of CRE3 mice containing either wild-type *casp8* genes or homozygous floxed *casp8* genes (N*casp8* ^−/−^). PCR was performed using primers specific for *casp8* (top) or the *cre* transgene (bottom). Brain tissues included cortex (lanes #1), thalamus/basal ganglia (lanes #2), and brain stem (lanes #3). Arrows indicate the expected PCR products for unrecombined (fl/fl) at 650 bp, recombined *caspase8* gene knockout at ∼200 bp (−/−), and wild-type unfloxed (+/+) c*asp8* genes at ∼800 bp. (**C**) SDS-PAGE immunoblot analysis of caspase 8 protein levels in mouse brain tissues. Brain samples from cortex (C), cerebellum (CB) or basal ganglia (BG) of *casp8^fl/fl^*-CRE3 mice were compared with CRE3 and *casp8^fl/fl^* animals. Lysates were normalized for total protein content (30 µg/lane) and analyzed by SDS-PAGE/immunoblotting using antibodies specific for mouse caspase 8 (top), or β-actin (bottom), using a multiple antigen detection method [39]. (**D–K**) Examples are provided of immunostaining for mouse caspase 8 in the brains of CRE3 (**D–G**) and N*casp8* ^−/−^ mice (**H–K**). Antibody detection was accomplished using diaminobenzidine (DAB) chromogen (brown); nuclei were counterstained with hematoxylin (blue). Magnification scale bar = ∼100 µm.

To establish mice with neuron-specific *caspase 8* gene ablation, we bred *casp8*-floxed (*casp8^fl/fl^*) mice with transgenic pan-neuronal CRE3 mice that express the cre protein exclusively in neurons [21]. Consecutive breeding of heterozygotes resulted in establishment of neuron-specific *casp8* knockout mice, henceforth termed N*casp8* ^−/−^. Genotypes were confirmed by PCR (Figure 1A). Genomic DNA was analyzed from various tissues to determine whether CRE3 induced recombination of the floxed-*casp8* gene in a tissue-specific manner (Figure 1B). PCR primers were designed to differentiate unrecombined and recombined floxed-*casp8*, as well as the wild-type murine *casp8* gene. Specimens from brain (cortex, thalamus/basal ganglia, brain stem) but not from other organs of the N*casp8* ^−/−^ mice and not from CRE3 (*cre/casp8^+/+^*) mice showed the expected PCR product of 200 bp indicative of brain-specific recombination (Figure 1B). Immunoblot analysis revealed decreased pro-caspase 8 levels in lysates from various regions of brain, including cortex (C), basal ganglia (BG), and cerebellum (CB) of the N*casp8* ^−/−^ mice compared to those from CRE3 (*cre/casp8^+/+^*) or *casp8*-floxed (*casp8^fl/fl^*) controls (Figure 1C), confirming that our genetic manipulations resulted in reduced caspase 8 expression in brain. Because the *cre* transgene employed is expressed only in neurons, glial expression of caspase 8 is expected to remain [42], possibly accounting for the residual pro-caspase 8 protein detected. Using immunohistochemistry (IHC), brains derived from control mice (Figure 1D–G) exhibited clear caspase 8 immunostaining in neurons and fibrous astroglia, whereas negligible caspase 8 staining was observed in neurons in brains from the N*casp8* ^−/−^ animals (Figure 1H–K). No gross or histological brain malformations were detected in the N*casp8* ^−/−^ mice.

### *Casp8*-ablated cultured embryonic neurons show resistance to TNFα-induced cell death

**Figure 2:**
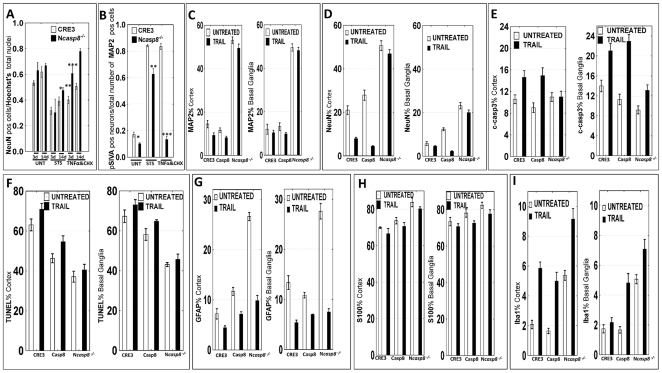
Neuroprotective effects of caspase 8 gene ablation demonstrated for cultured primary neurons. (**A**) The ratio of surviving NeuN-positive neurons that showed Hoechst staining profile characteristic of viable cells to the total cell number (number of Hoechst-positive nuclei) was determined after 3 and 14 days of culture for untreated (UNT), staurosporine- (STS) and TNFα/CHX-treated primary cortical neurons derived from embryos (E16.5) of CRE3 and N*casp8* ^−/−^ homozygous mice. Cells were counted on ten random fluorescent images (×20). (**B**) The ratio of pSIVA-positive neurons with colocalized MAP2 expression to the total number of MAP2-positive cells was determined after 14 days of culture for UNT, STS- and TNFα/CHX-treated primary cortical neurons derived from embryos of CRE3 and N*casp8* ^−/−^ mice. Cells were counted on 15 random fluorescent images (×20) from 3 experiments. (**C–I**) Bar graphs present average percentages of MAP2 (**C**), NeuN (**D**), cleaved caspase 3 (**E**), GFAP (**G**), S100 (**H**), Iba1 (**I**) immunopositive staining and mean percentage of TUNEL positive signaling (**F**) in cortices (left) and basal ganglia (right) of untreated and TRAIL-treated cultured coronal brain slices derived from CRE3, *casp8^fl/fl^* (Casp8 line) and N*casp8* ^−/−^ mice after 3 days of culture. **Tables 1** **and** **2** provide the results of statistical analyses.

**Figure 3:**
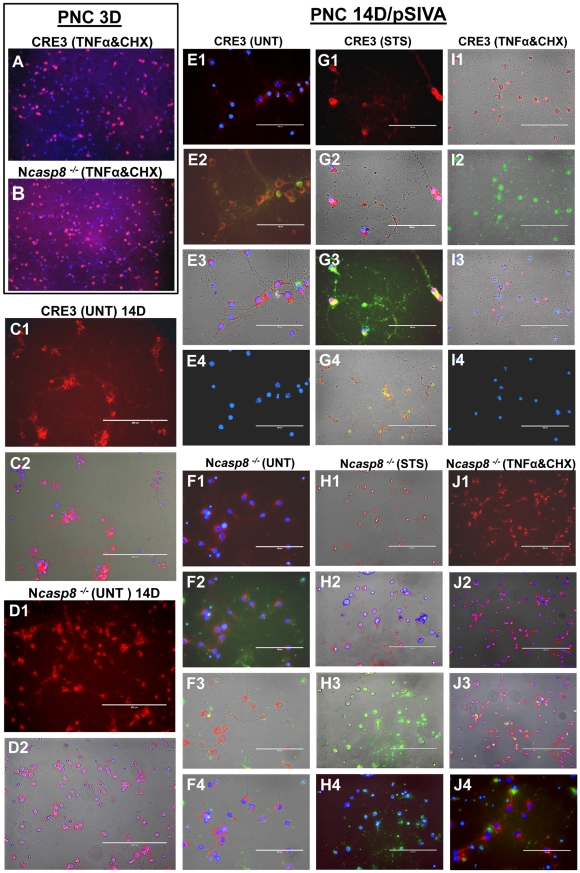
Caspase 8 gene ablation provides neuroprotection in vitro in primary neuronal cultures and brain organotypic cultures. (**A–B**) 3-day-old PNC derived from embryos (E16.5) of CRE3 (**A**) and N*casp8* ^−/−^ homozygous mice (**B**) were treated with recombinant murine TNFα and cycloheximide (CHX), then fixed and stained with Hoechst dye and immunostained with an antibody to NeuN followed by application of Alexa Fluor 594 conjugated anti-mouse antibody. (**C1–J4**). Untreated (UNT **C1–F4**), STS-treated, (**G1–H4**) and recombinant murine TNFα and CHX-treated (**I1–J4**) 14-day-old neuronal cultures derived from CRE3 and N*casp8* ^−/−^ mice were preincubated with pSIVA reagent (green) for 15 min prior to Z-fix fixation, then immunostained for MAP2 (red) and stained with DAPI (blue). To assess neuronal phenotype, the transmission light, single (**C1, D1, E4, G1, H1, H3, I1, I2, I4, J1**), double (**C2, D2, E1, E2, F1, F3, G4, H2, H4, I3, J2**), and triple (**E3, F2, F4, G2, G3, J3, J4**) channel images are provided. Colocalization of neuronal marker MAP2 (red) with pSIVA signal (green) resulted in yellow color denoting degenerating neurons whose number was counted and plotted as ratio to the total number of neurons (**Figure 2B**). Single and multichannel panels confirm specificity of the double labeling. PSIVA was visualized from the early (membranous staining) to later stages (staining in the cell body and nucleus, the latter in cells with loss of plasma cell integrity) of neuronal degeneration.

To functionally verify the effects of caspase 8 deletion in murine neurons, cultured embryonic cortical neurons from the CRE3 and N*casp8* ^−/−^ homozygous mice were maintained *in vitro* for 3 and 14 days prior to further experimentation. Comparison of CRE3 and N*casp8* ^−/−^ neurons after 14 days of culture revealed significant differences in their phenotype and survival rates (Figure 2A, B; Figure 3C1–D2). In contrast to sparsely distributed, clustered control (CRE3) neurons (Figure 3C1, C2), cultures derived from N*casp8* ^−/−^ homozygous mice formed dense networks of highly branched interconnected neurons (Figure 3D1, D2). Double staining for MAP2 (red) and pSIVA (green) [27]confirmed this observation, revealing decreased proportion of spontaneously degenerating neurons in the N*casp8* ^−/−^ culture compared to the CRE3 control (Figure 2B, Figure 3E1–F4). After 3 and 14 days in culture, cell death was induced using either TNFα plus cycloheximide, an agonist of the death extrinsic apoptosis pathway (death receptor), or staurosporine, a broad-spectrum protein kinase inhibitor that induces the intrinsic (mitochondrial) pathway of cell death. Consistent with a prominent role for caspase 8 in the extrinsic pathway, *casp8* gene deletion significantly protected immature and mature neurons against cell death induced by TNFα plus cycloheximide (Figure 2A **p = 0.03, B ***p<0.0001; Figure 3A, B, I1–J4), as determined by the ratio of remaining (surviving) NeuN-positive neurons with Hoechst staining profile characteristic of viable cells, relative to total cell number (number of Hoechst-positive nuclei) (Figure 3A). Partial protection against staurosporine-induced cell death was observed only for mature N*casp8* ^−/−^ neurons (*p = 0.004) but not for the immature neuronal cells (Figure 2A). PSIVA staining of the mature neurons confirmed this observation, demonstrating a reduced proportion of apoptotic/degenerating neurons in the STS-treated and also in untreated and TNFα/CHX-treated N*casp8* ^−/−^ neurons compared to the controls (**p = 0.0001; *p = 0.004; ***p<0.0001) (Figure 2B; Figure 3E1–J4). This tendency was particularly apparent in the TNFα plus cycloheximide-treated neurons, where preserved neuronal morphology in the N*casp8* ^−/−^ cultures contrasted with the presence of devastating neuronal degeneration with loss of axonal and dendritic branches, and numerous apoptotic bodies in the CRE3 cultures (Figure 3I1–J4). We conclude that homozygous deletion of genes encoding caspase 8 in neurons significantly protects immature and mature neuronal cells against cell death induced *via* the extrinsic apoptosis pathway, thus functionally validating the genotypic and expression data above.

### Neuronal deletion of *casp8* protects neurons in an organotypic culture model

In addition to assessing the impact of caspase 8 deletion in explanted monolayer cultures of fetal neurons, we also evaluated brain coronal slices from adult N*casp8* ^−/−^ mice compared to control animals. Although brain slice cultures are well-established models for *in vitro* investigations, they have been typically prepared from early postnatal rodent brains, which show a high plasticity and resistance to mechanical trauma during the slice preparation, thus maintaining longer viability in culture (reviewed in [43]) [44]. In contrast, our brain slices are derived from 4–8 month old adult mice. Although preparation of slice cultures from older animals is more challenging, the model is more suitable to study pathology of the mature brain [45], [46]. Unlike organotypic slices encompassing restricted brain regions, we have deliberately prepared whole brain slices to study cell death of neurons in mice with pan-neuronal expression of *cre* recombinase, in *casp8^fl/fl^* line, and mice with conditional deletion of *casp8* in neurons.

**Figure 4:**
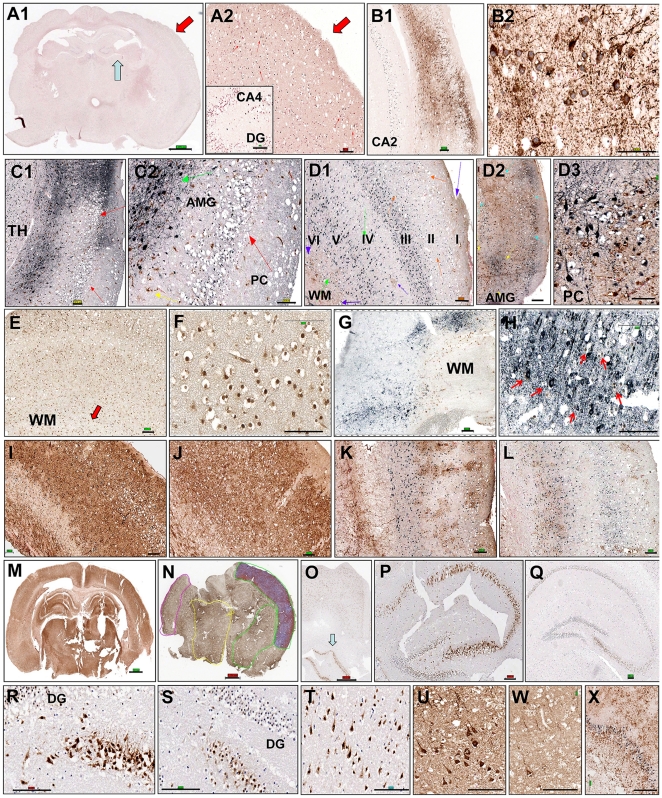
Casp8 ablation provides neuroprotection in organotypic brain slice cultures. The coronal brain slices derived from the CRE3 control line and N*casp8* ^−/−^ homozygous mice were maintained in *in vitro* conditions for 3 (**A1–M**) and 14 days (**N–X**). The presented results were obtained from untreated (**A1–D3, I, K, N–X**) and TRAIL-treated (**E–H, J, L**) cultures. Immunohistochemical stainings are depicted in cortical and subcortical regions of CRE3 (**A1, A2, E, F, Q, S, W**) and N*casp8* ^−/−^ (**B1–D3, G–P, R, T, U, X**) brain slices. Examples of single marker (**M–W**) and double (**A1–L, X**) immunostainings are presented for S100 (**M, N**), NeuN (**O–T**), and β3-tubulin (**U, W**) as well as for double labeled MAP2 (DAB-brown) and cleaved caspase 3 (SG-black; **A1–B2**) or GFAP (DAB; **D1–D3**), TUNEL (DAB; **E–H**) with MAP2/NeuN antibody cocktail (SG) and S100 (DAB; **I–J**); IbA1 (DAB; **C1–C2**) or GFAP (DAB; **D1–D3**) with NeuN (SG) immunostaining. Scale bar indicates ∼1000 µm on macro images and ∼100 µm on high magnification images of the slices. Abbreviations: **DG** = Dental Gyrus; **CA2; CA4** = sectors of hippocampus; **TH** = Thalamus; **AMG** = Amygdala; **PC** = Piriformis Cortex; **WM** = White Matter.

The aim of the present study was to determine the preservation of neurons in mature brain slices maintained *in vitro* for 72 h and 14 days. First, we have analyzed “spontaneous” neuronal cell death following slice preparation in the 3-day-old culture. The insults associated with this technique, including tissue injury (trauma), loss of cerebral blood flow (with likely ischemia/hypoxia and hypoglycemia), and denervation (axotomy) are known to induce cell death. Microscopic analysis of paraffin-embedded brain slices by H&E and Masson's trichrome staining revealed various degrees of cellular degenerative and edematous changes, particularly pronounced in the hippocampi and deep basal ganglia. The survival of neurons in brain slices, however, was maintained significantly better in the knockout compared to control mice. The percentages of surviving neurons in brain slices were evaluated in various regions of the brain by MAP2 and neuron-specific nuclear protein (NeuN) immunostaining. MAP2 protein localizes predominantly to the dendrites and perikaryon of intact neurons and is disrupted by neuronal damage [47]. As assessed by MAP2 or NeuN immunostaining, spontaneous loss of neurons in brain slices was highly pronounced in tissue sections derived from CRE3 or *casp8^fl/fl^* mice (Figure 2C, D; Figure 4A1–A2), whereas significantly more surviving neurons were present in brain slices from N*casp8* ^−/−^ animals (Figure 2C, D; Figure 4B1–B2; Table 1). The decline in MAP2 protein expression was associated with increased levels of cleaved caspase 3 (Figure 4A1, A2; A2 inset – high magnification micrograph of the primary motor cortex and hippocampus indicated by red and blue arrows).

**Table 1:** Analysis of TUNEL assay and protein biomarkers in cortical and basal ganglia regions of untreated and TRAIL-treated coronal brain slices (3-day-old BOCSC).

MOUSE LINES &	TUNEL	c-*casp*3	MAP2	NeuN	GFAP	S100	Iba1
BRAIN REGIONS	(mean±SEM); *p* value	(mean±SEM); *p* value	(mean±SEM); *p* value	(mean±SEM); *p* value	(mean±SEM); *p* value	(mean±SEM); *p* value	(mean±SEM); *p* value
**CORTEX: Untreated ** ***vs* TRAIL-Treated**							
**CRE3**	63±2.9 *vs* 71±3.0; *p* NS	10±0.4 *vs* 15±1.2; *p* = 0.03	14±1.7 *vs* 9±1.1; *p* = 0.03	21±2.0 *vs* 8±0.6; *p* = 0.0002	7±1.0 *vs* 4±0.4; *p* = 0.03	70±0.4 *vs* 67±2.8; *p* NS	2±0.3 *vs* 6±1.04; *p*<0.0001
***casp*** **8^fl/fl^**	46±2.3 *vs* 55±2.9; *p* NS	9±0.9 *vs* 15±1.4; *p* = 0.007	11±1.1 *vs* 8±0.7; *p* = 0.03	28±2.3 *vs* 4±0.3; *p*<0.0001	12±0.8 *vs* 7±0.5; *p* = 0.0001	74±1.8 *vs* 71±2.1; *p* NS	1.7±0.2 *vs* 5±0.6; *p* = 0.0006
**N** ***casp*8^−/−^**	37±2.7 *vs* 41±2.8; *p* NS	11±0.8 *vs* 11±1.1; *p* NS	53±1.5 *vs* 49±1.9; *p* NS	51±2.3 *vs* 47±1.9; *p* NS	27±0.8 *vs* 10±1.04; *p*<0.0001	84±2.4 *vs* 80±1.1; *p* NS	5±0.3 *vs* 9±0.7; *p* = 0.0001
**BASAL GANGLIA: Untreated ** ***vs* TRAIL-Treated**							
**CRE3**	67±3.1 *vs* 73±2.6; *p* NS	14±1.2 *vs* 21±1.5; *p* = 0.006	12±2.3 *vs* 10±1.1; *p* NS	6±0.8 *vs* 5±0.3; *p* NS	13±1.4 *vs* 5±0.5; *p* = 0.0006	74±2.3 *vs* 71±1.6; *p* NS	1.7±0.3 *vs* 2±0.3; *p* NS
***casp*** **8^fl/fl^**	58±2.9 *vs* 65±0.7; *p* NS	11±1.1 *vs* 23±1.4; *p* = 0.0002	13±1.7 *vs* 10±0.7; *p* NS	12±0.7 *vs* 2±0.2; *p*<0.0001	11±0.6 *vs* 7±0.1; *p* = 0.0001	78±2.3 *vs* 73±1.3; *p* NS	2±0.3 *vs* 5±0.6; *p* = 0.002
**N** ***casp*8^−/−^**	43±1.0 *vs* 46±2.7; *p* NS	9±0.8 *vs* 13±1.1; *p* = 0.02	50±1.7 *vs* 48±1.4; *p* NS	23±1.4 *vs* 20±1.2; *p* NS	28±1.6 *vs* 8±0.6; *p*<0.0001	82±1.6 *vs* 78±2.3; *p* NS	5±0.3 *vs* 7±0.6; *p* = 0.02
**CORTEX: Untreated**							
**CRE3 ** ***vs* N*casp*8^−/−^**	63±2.9 *vs* 37±2.7; *p* = 0.0002	11±0.8 *vs* 11±0.8; *p* NS	14±1.7 *vs* 53±1.5; *p*<0.0001	21±2.0 *vs* 51±2.3; *p*<0.0001	7±1.0 *vs* 27±0.8; *p*<0.0001	70±0.4 *vs* 84±2.4; *p* = 0.0004	2±0.3 *vs* 5±0.3; *p*<0.0001
***casp*** **8^fl/fl^*vs* N*casp*8^−/−^**	46±2.3 *vs* 37±2.7; *p* = 0.03	9±0.9 *vs* 11±0.8; *p* NS	12±1.1 *vs* 53±1.5; *p*<0.0001	28±2.3 *vs* 51±2.3; *p* = 0.0001	12±0.8 *vs* 27±0.8; *p*<0.0001	74±1.8 *vs* 84±2.4; *p* = 0.009	1.7±0.2 *vs* 5±0.3; *p*<0.0001
**CORTEX: TRAIL-Treated**							
**CRE3 ** ***vs* N*casp*8^−/−^**	71±3.0 *vs* 41±2.8; *p* = 0.00007	15±1.2 *vs* 11±1.1; *p* = 0.06 (NS)	9±1.1 *vs* 49±1.9; *p*<0.0001	5±0.3 *vs* 47±1.9; *p*<0.0001	4±0.4 *vs* 10±1.04; *p* = 0.001	67±2.8 *vs* 80±1.1; *p* = 0.002	6±1.04 *vs* 9±0.7; *p* = 0.004
***casp*** **8^fl/fl^*vs* N*casp*8^−/−^**	55±2.9 *vs* 41±2.8; *p* = 0.008	15±1.4 *vs* 11±1.1; *p* = 0.06 (NS)	8±0.7 *vs* 49±1.9; *p*<0.0001	4±0.3 *vs* 47±1.9; *p*<0.0001	4±0.4 *vs* 10±1.04; *p* = 0.047	74±1.8 *vs* 80±1.1; *p* = 0.003	1.7±0.2 *vs* 9±0.7; *p* = 0.002
**BASAL GANGLIA: Untreated**							
**CRE3 ** ***vs* N*casp*8^−/−^**	67±3.1 *vs* 43±1.0; *p* = 0.00008	14±1.2 *vs* 9±0.8; *p* = 0.01	12±2.3 *vs* 50±1.7; *p*<0.0001	6±0.8 *vs* 23±1.4; *p*<0.0001	13±1.4 *vs* 28±1.6; *p* = 0.0001	74±2.3 *vs* 82±1.6; *p* = 0.01	1.7±0.3 *vs* 5±0.3; *p*<0.0001
***casp*** **8^fl/fl^*vs* N*casp*8^−/−^**	58±2.9 *vs* 43±1.0; *p* = 0.001	11±1.1 *vs* 9±0.8; *p* NS	13±1.7 *vs* 50±1.7; *p*<0.0001	12±0.7 *vs* 23±1.4; *p* = 0.0001	11±0.6 *vs* 28±1.6; *p*<0.0001	78±2.3 *vs* 82±1.6; *p* NS	2±0.3 *vs* 5±0.3; *p*<0.0001
**BASAL GANGLIA: TRAIL-Treated**							
**CRE3 ** ***vs* N*casp*8^−/−^**	73±2.6 *vs* 46±2.7; *p* = 0.00008	21±1.5 *vs* 13±1.1; *p* = 0.003	10±1.1 *vs* 48±1.4; *p*<0.0001	5±0.3 *vs* 20±1.2; *p*<0.0001	5±0.5 *vs* 8±0.6; *p* = 0.03	71±1.6 *vs* 78±2.3; *p* = 0.04	2±0.3 *vs* 7±0.6; *p* = 0.0001
***casp*** **8^fl/fl^*vs* N*casp*8^−/−^**	65±0.7 *vs* 46±2.7; *p* = 0.0001	23±1.4 *vs* 13±1.1; *p* = 0.0006	10±0.7 *vs* 48±1.4; *p*<0.0001	2±0.2.0 *vs* 20±1.2; *p*<0.0001	7±0.1 *vs* 8±0.6; *p* NS	73±1.3 *vs* 78±2.3; *p* NS	5±0.6 *vs* 7±0.6; *p* = 0.04

Statistical analyses of control (**CRE3**, ***casp8^fl/fl^***) *vs* **N** ***casp8*** **^−/−^** mouse specimens were performed using Student's t-test. The resulting average percentages of positive/immunopositive cells and standard error of mean (SEM) values are provided. *P* values<0.05 are reported as statistically significant. “NS” stands for nonsignificant (*p*≥0.05).

Investigation of the entire coronal brain slices at 72 h provided information about regional vulnerability of neurons and the dynamics of spontaneous neuronal degeneration in organotypic culture. Immunostaining revealed a relatively early loss of MAP2 immunoreactivity, suggesting rapid vulnerability of the microtubular cytoskeleton, whereas more robust immunoreactive signals for NeuN were detected even in neurons with balloon degeneration (pointed to by the red arrows in Figure 4C1, C2). In normal mice (that contain intact caspase 8 genes), degeneration and death of neurons occurred during the first 24 h in the entire brain. In brain slices derived from N*casp8* ^−/−^ mice, the most susceptible regions were entorhinal cortex and hippocampus, whereas neurons in basal ganglia (particularly in the amygdala and the bed nucleus of the stria terminalis) maintained well-preserved morphology and MAP2 immunoreactivity for up to 3 days in culture (Figure 4B1–D3; green arrows). Also, the median preoptic nucleus, the dorsal part of caudate putamen, median and lateral habenular nuclei with adjacent parafascicular thalamic nucleus, and posterior thalamic nuclear group contained well-preserved neurons. In neocortex of the N*casp8* ^−/−^ slices, neurons in superficial cortical layers (I–II) were more affected (Figure 4D1; red arrows), whereas deeper cortical layers (III–VI) were less impacted by organotypic culture conditions (Figure 4D1; green arrows). Similar regional susceptibility had been suggested in prior reports for cultures of dissociated cortical layers [48].

Considering that at least part of neuronal cell death that occurs in the brain slice culture model is due to signals produced by tissue injury, we speculated that cytokines are elaborated locally in the sliced brain tissue, resulting in caspase 8 activation. As an example, we used the TNF-related apoptosis-inducing ligand (TRAIL) to evoke cell death in the cultured brain slices. TRAIL is known to transmit the apoptotic signals through death receptors that directly activate caspase 8 [49]. Interestingly, expression of TRAIL-binding death receptors is induced by the transcription factor CHOP during ER stress, a condition associated with oxidative stress and ischemia-reperfusion injury (reviewed in [50]). In the brain organotypic culture model, *casp8* gene deletion significantly protected neurons against cell death induced by TRAIL, as estimated in the cortical region and basal ganglia. In contrast to control brain slices from CRE3 and *casp8^fl/fl^* mice where TRAIL reduced the prevalence of MAP2 and NeuN neurons (Figure 4E, F), no significant difference was observed in the number of cells expressing neuronal markers following TRAIL treatment of *Ncasp8* ^−/−^ slices (Figure 2C, D; Figure 4G, H; Table 1). Deletion of *casp8* offered partial protection against TRAIL-induced apoptosis in organotypic cultures, as demonstrated by significantly decreased percentages of cleaved caspase 3- and TUNEL-positive cells in the *Ncasp8* ^−/−^ compared to CRE3 and *casp8^fl/fl^* brain slices (Figure 2C, D, F; Table 1). Figure 4 (E–H) demonstrates representative images of a reciprocal pattern of TUNEL and MAP2 expression in brain slices derived from the control line and *Ncasp8* ^−/−^ mice.

Not only did TRAIL induce apoptosis of caspase 8-containing neurons in organotypic brain slice cultures, but some types of glial cells also appeared to be vulnerable to treatment with this cytokine in the organotypic brain slice setting. In this regard, TRAIL treatment significantly decreased the number of GFAP-positive fibrous astroglia in all three genotype groups (Figure 2G), suggesting that these glial cells are sensitive to TRAIL and serving as a control to show the selective protection of neurons in *Ncasp8* ^−/−^ slices (Figure 4K, L). Interestingly, the percentage of S100-positive protoplasmic astrocytes did not change following TRAIL treatment (Figure 2H; Figure 4I, J), implying that these glial cells are insensitive to TRAIL under the culture conditions tested (Table 1). Immunostaining for the microglial marker Iba1 increased after TRAIL treatment in brain slice culture derived from all 3 genotypes (Figure 2I), serving as a further indicator of the neuron-specific differences in the responses of *Ncasp8* ^−/−^ slices to TRAIL treatment and a sign of viability of stromal cells in the brain *in vitro*. After 14 days of culture, untreated *Ncasp8* ^−/−^ brain slices demonstrated significantly more surviving neurons compared to the controls as evidenced by higher levels of NeuN protein in the cerebral cortices (Table 2 *p* = 0.0004; Figure 4O, T in neocortex; Figure 4O – arrow, P, R in archicortex/hippocampus), in basal ganglia (Figure 4O) and higher prevalence of microtubule-associated class III β-tubulin in gray matter, particularly in basal ganglia of the *Ncasp8* ^−/−^ brains (Table 2 *p* = 0.0009; Figure 4U, W). MAP2 protein was undetectable in the control slices and it was found only in neuropil but not in neuronal soma in the N*casp8* ^−/−^ cultures. Activation of astro- and microglia was more pronounced in *Ncasp8* ^−/−^ brain slices than in the controls, as demonstrated by higher percentage of GFAP (Table 2 *p* = 0.001 in basal ganglia; Figure 4X) and Iba1 (Table 2 *p* = 0.0004 in cortex; *p* = 0.0003 in basal ganglia) immunostainings. Levels of S100 protein, that visualizes protoplasmic astroglia, were comparable at different time points for all three lines, as presented for N*casp8* ^−/−^ slices at day 3 and 14 in culture (Figure 4M and N, respectively).

**Table 2:** Analysis of TUNEL assay and protein biomarkers in cortical and basal ganglia regions of untreated 14-day-old organotypic brain slice cultures (BOCSC).

MOUSE LINES &	TUNEL	c-*casp*3	MAP2	NeuN	β3 tubulin	GFAP	S100	Iba1
BRAIN REGIONS	(mean±SEM); *p* value	(mean±SEM); *p* value	(mean±SEM); *p* value	(mean±SEM); *p* value	(mean±SEM); *p* value	(mean±SEM); *p* value	(mean±SEM); *p* value	(mean±SEM); *p* value
CORTEX:								
**CRE3 ** ***vs* N*casp*8^−/−^**	72±2.4 *vs* 60±8.0; *p* NS	41±6.6 *vs* 53±7.9; *p* NS	16±1.7 *vs* 13±2.7; *p* NS	32±1.4 *vs* 57±4.5; *p* = 0.0004	39±4.5 vs 44±3.3; *p* NS	21±4.9 *vs* 25±2.1; *p* NS	51±2.7 *vs* 49±1.6; *p* NS	51±2.7 *vs* 49±1.6; *p* = 0.0004
**BASAL GANGLIA:**								
**CRE3 ** ***vs* N*casp*8^−/−^**	68±1.8 *vs* 62±5.9; *p* NS	29±5.5 *vs* 39±4.9; *p* NS	15±1.4 *vs* 11±1.9; *p* NS	16±3.9 *vs* 24±3.1; *p* NS	27±1.6 *vs* 24±3.1 *p* = 0.0009	15±1.7 *vs* 26±1.7; *p* = 0.001	53±5.8 *vs* 57±2.0; *p* NS	8±0.9 *vs* 20±2.0; *p* = 0.0003

Statistical analyses of control **CRE3** *vs* **N** ***casp8*** **^−/−^** mouse specimens were performed using Student's t-test. The resulting average percentages of positive/immunopositive cells and standard error of mean (SEM) values are provided. *P* values<0.05 are reported as statistically significant. “NS” stands for nonsignificant (*p*≥0.05).

### Neuronal deletion of caspase 8 protects against traumatic brain injury and facilitates learning and memory retention in aged mice

**Figure 5:**
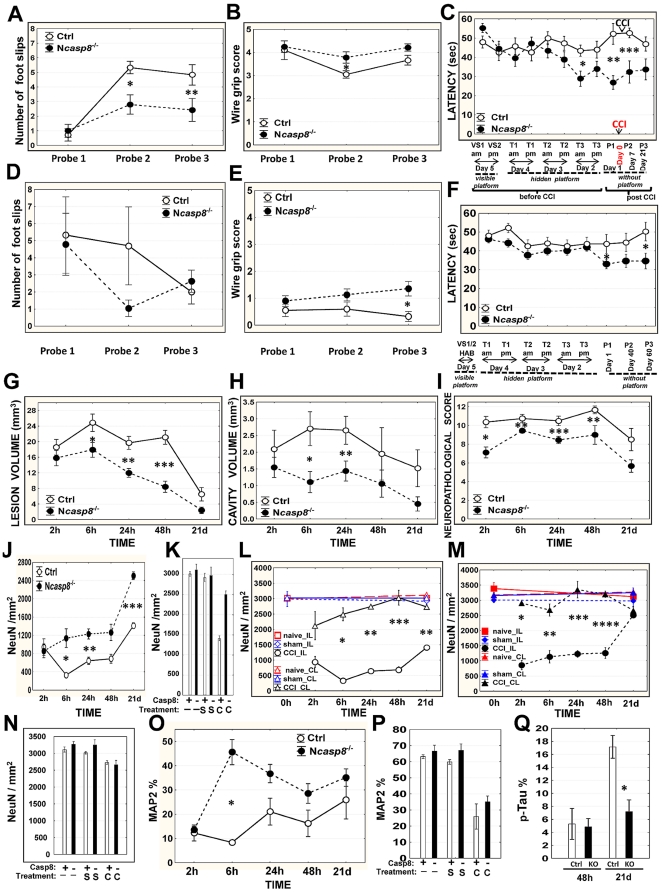
Caspase 8 deficiency affects functional and histopathological outcome following TBI and facilitates learning and memory retention in aged mice. (**A**) Sensorimotor function was measured as the number of foot slips while traversing the length of the beam three times in the beam walking trial. Training was followed by probe 1 (one week interval) before CCI procedure and repeated 7 and 21 days after the procedure (probe 2 and 3). *P* values result from Student's t-test (*p = 0.009; **p = 0.04). (**B**) Wire grip scores were quantified using a 5-point scale [33] to assess motor function. Tests were conducted 1 day before CCI (probe 1) and 7 (probe 2; *p = 0.03) and 21 days (probe 3) after brain trauma. (**C**) Morris water maze (MWM) performance was tested in the control (Ctrl; n = 12) and N*casp8* ^−/−^ (n = 15) mice. Average latency to reach and climb the platform or enter the platform zone before and after CCI is shown (mean±SEM) (*p = 0.005; **p = 0.001; ***p = 0.009). Beam walking (**D**) and wire grip (**E**) tests were conducted in uninjured 18 month old *casp8^fl/fl^* (Ctrl; n = 19) and N*casp8* ^−/−^ (n = 20) mice (3 testing sessions, spaced one week apart). *P* values result from Student's t-test (*p = 0.01). (**F**) MWM task was performed in the same aged cohort to test memory retention up to 60 days after completion of the learning sessions. Average latency to reach and climb the platform is shown (mean±SEM) (*p = 0.04). (**G–I**) Histopathological outcomes were compared between the control (Ctrl) and N*casp8* ^−/−^ mice 2 h, 6 h, 24 h, 48 h, and 3 weeks after the trauma (8–10 mice per time point in each experimental group). Using Aperio scanning system, lesion (**G**) and cavity (**H**) volumes were determined by summing each lesion or cavity area multiplied by the distance between each coronal slice (mean±SEM). Plots depict average lesion volume (**G**: *p = 0.03; **p = 0.002; ***p = 0.003), cavity volume (**H**: *p = 0.02; **p = 0.03), and neuropathological scores (**I**: *p = 0.002; **p = 0.02; ***p = 0.004). (**J**) Time course for density (n/mm^2^) of NeuN-immunopositive neurons in traumatic brain lesions is depicted for the control (Ctrl) and N*casp8* ^−/−^ mice. (**K**) The NeuN immunostaining results are compared among the six experimental groups [control (Ctrl) and N*casp8* ^−/−^ naïve (no surgery “-”), sham (surgery “S”), and subjected to CCI (“C”) mice] in impact lesions (CCI-treated) or corresponding regions (naïve or sham-operated) at 21 day after procedure. Density (n/mm^2^) of NeuN-immunopositive neurons in impact lesions or matching ipsilateral regions (IL) and corresponding contralateral (CL) regions of the control (Ctrl) (**L**) and N*casp8* ^−/−^ (**M**) naïve (no surgery “-”), sham (surgery “S”), and subjected to CCI (“C”) mice is presented in a time-course manner (**L, M**) or at 21 day after procedure (**N**) (**J**: *p = 0.02; **p = 0.01; ***p = 0.0004; **L**: *p = 0.0005; **p<.0001; ***p = 0.0008; **M**: *p = 0.0002; **p = 0.006; ***p = 0.002; ****p = 0.0007). (**O–P**) Percentages of MAP2 immunopositive neurons were compared in impact lesions or corresponding ipsilateral regions of the control (Ctrl) and N*casp8* ^−/−^ naïve (no surgery “-”), sham (surgery “S”), and subjected to CCI (“C”) mice (8–10 mice/time point/group) and presented as time-course data (**O**) at 5 time points post CCI (mean±SEM) (**L**: *p = 0.002) and at 21 day after CCI (bar graph)(**P**). (**Q**) Phospho-tau immunoreactivity was assessed in ipsilateral semihemispheres of the control and N*casp8* ^−/−^ mice 48 h and 3 weeks after CCI (n = 8–10 per group) (mean ± SEM *p = 0.001).

To assess the effect of caspase 8 neuronal knockout on functional performance and histopathological outcome following brain injury, we subjected mice to TBI using a CCI model. Comparisons were made of N*casp8* ^−/−^, CRE3 (*cre/casp8^+/+^*), *casp8*-floxed mice (*casp8^fl/fl^*), and wild-type littermates (*casp8^+/+^*). All knockout (n = 135) and 152 of 160 (95%) control animals survived CCI procedure and selected groups were put through behavioral tests. Sensorimotor function was evaluated by beam walking and hindlimb flexion tests. All naïve control and knockout mice learned to balance on the wooden beam (Figure 5A; probe 1). Although all mice were impaired in their performance after CCI, brain-injured N*casp8* ^−/−^ mice performed significantly better at this task (probe 2, 3; *p = 0.009; **p = 0.04), demonstrating fewer foot slips during crossing compared to their control counterparts. In the hindlimb flexion test, none of the tested mice showed movement asymmetry or paresis prior to the CCI procedure (probe 1). Following the brain trauma, 6 of 7 (86%) control *vs* 2 of 7 (29%) N*casp8* ^−/−^ mice (probe 2; Chi-square *p* = 0.03), and 6 of 7 (86%) control *vs* 1 of 7 (14%) N*casp8* ^−/−^ animals (probe 3; Chi-square *p* = 0.008) showed neurological deficits. To assess motor function, we used the wire grip test. N*casp8* ^−/−^ mice performed better than the control cohort (Figure 5B), with statistically significant differences observed between groups at 7 days after CCI (probe 2 *p = 0.03). To determine the effect of neuronal caspase 8 ablation on spatial memory acquisition, mice were subjected to Morris water maze (MWM) test before and after CCI. N*casp8* ^−/−^ mice demonstrated better learning performance to locate the hidden platform based on visual cues, as well as superior memory retention following the cued learning phase prior to CCI and significantly shorter latencies after CCI compared to the control animals (T3 *p = 0.005; probe 1 **p = 0.001; probe 2 ***p = 0.009) (Figure 5C).

Better performance of N*casp8* ^−/−^ mice in the wire grip and MWM tests compared to control animals was evident also in a cohort of aged (18 month old; *casp8^fl/fl^* n = 19, and N*casp8* ^−/−^ n = 20) mice with no brain injury (Figure 5E *p = 0.01; Figure 5F *p = 0.04, respectively), although the wire grip test revealed a dramatic age-dependent decline in motoric performance (p<0.0001 – probes 1–3) in all lines. Behavior of aged mice in the beam walking test did not reveal statistically significant differences between the genotypes (Figure 5D).

### Protective effects of neuronal caspase 8 deletion on brain histopathology after CCI

**Figure 6:**
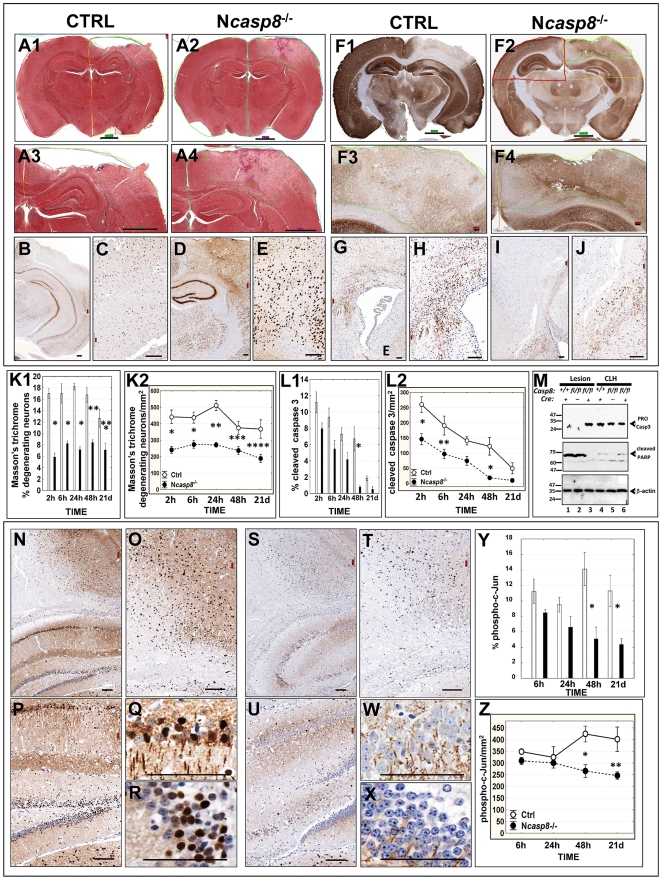
Assessment of lesion volume, cell death and immunoreactivity for MAP2, NeuN, and phospho-tau in control and Ncasp8^−/−^ brains after CCI. (**A1–A4**) Masson's trichrome–stained brain sections were digitalized using the Aperio scanning system. Digitized virtual slides were annotated by encircling the brain tissue loss (cavity) and lesion edges (penumbra) between intact and pathologically changed brain tissue of the control (**A1, A3**) and N*casp8* ^−/−^ (**A2, A4**) mice. Scale bar = ∼1000 µm on low and ∼100 µm on high magnification images. Examples of nuclear NeuN (**B–E**) and the perikaryonal and dendritic MAP2 (**F1–F4**) immunostaining are presented in the coronal brain sections (**F1, F2**), cortical lesions and adjacent structures (**B–E, F3, F4**) of the control (**B, C, F1, F3**) and N*casp8* ^−/−^ (**D, E, F2, F4**) mice after CCI. Scale bar = ∼1000 µm on low (**F1, F2**) and ∼100 µm on higher magnification images (**B–E, F3, F4**). Examples of phospho-tau immunostaining in control (**G, H**) and N*casp8* ^−/−^ (**I, J**) brains are provided at low and high-power magnifications (scale bar = ∼100 µm). Using Aperio software, percentage (**K1, L1**) and density (n/mm^2^) (**K2, L2**) of degenerating (**K1,2**) and apoptotic (**L1,2**) neurons was identified by Masson's trichrome stain and cleaved caspase 3 immunostaining, respectively (mean±SEM; n = 8–10 per group and per time point) in lesion areas of brains from CRE3 (white bar) and N*casp8* ^−/−^ (black bar) mice at 2 h, 6 h, 24 h, 48 h, and 3 weeks after trauma (**K1**: *p = <.0001; **p = 0.003; ***p = 0.02, **K2**: *p = 0.0001; **p<.0001; ***p = 0.05; ****p = 0.01; **L1**: *p = 0.02; L2: *p = 0.02; **p = 0.03). (**M**) SDS-PAGE immunoblot analysis of caspase 3 and cleaved PARP protein levels in mouse brain tissues. Brain samples from impact lesion and corresponding region in the contralateral hemisphere (CLH) of N*casp8* ^−/−^ mice (lanes 3, 6) were compared with those from CRE3 (lanes 1, 4) and *casp8^fl/fl^* (lanes 2, 5) animals. Lysates were normalized for total protein content (30 µg/lane) and analyzed by SDS-PAGE/immunoblotting using antibodies specific for caspase 3, cleaved PARP, or β-actin, using a multiple antigen detection method [39]. (**N–X**) Examples are provided of phospho-c-Jun immunostaining in ipsilateral semi-hemispheres of brains from the control (**N–R**) and N*casp8* ^−/−^ (**S–X**) mice (magnification scale bar = ∼100 µm). Antibody detection was accomplished using polymer-based EnVision-HRP-enzyme conjugate (DakoCytomation) and diaminobenzidine (DAB) chromogen (brown); nuclei were counterstained with hematoxylin (blue). Percentage (**Y**) and density (**Z**) of phospho-c-Jun immunopositive nuclei were determined in impact lesions from control (white bar; Ctrl) and N*casp8* ^−/−^ (black bar) animals at 6, 24, 48 h, and 21 days post CCI (mean±SEM; n = 8–10 mice per group and per time point) (**Y**: *p = 0.03; **Z**: *p = 0.02; **p = 0.05).

In the brain-injured N*casp8* ^−/−^ mice, the average lesion volume was significantly reduced at 6 (*p = 0.03), 24 (**p = 0.002), and 48 h (***p = 0.003) after CCI compared to the control animals (Figure 5G). Whereas brain lesions of N*casp8* ^−/−^ mice were consistently confined to the ipsilateral cortex (Figure 6A2, A4), in caspase 8-expressing control animals the lesions often extended beyond the cortex, reaching the white matter or even the hippocampus (Figure 6A1, A3). Control animals also exhibited greater average cavity volumes at later time points post injury (6 h *p = 0.02; 24 h **p = 0.03) (Figures 5H and 6A1, A3). In addition to lesion and cavity volumes, brain injury scoring was performed, assessing neuropathological changes and anatomical extent of brain damage. Calculation of neuropathological scores revealed lower mean values in N*casp8* ^−/−^ mice compared to control animals (*p = 0.002; **p = 0.02; ***p = 0.004) (Figure 5I), corroborating the lesion and cavity volume data, thus further indicating that mice with neuronal caspase 8 deficiency suffer less severe brain injury following CCI as assessed by histopathological methods.

To assess neuronal preservation, a monoclonal antibody to NeuN was applied in IHC studies. Strongly expressed in normal neurons, NeuN immunoreactivity is diminished under pathological conditions, such as cerebral ischemia and brain trauma [51], [52], and proposed as a biomarker to predict delayed neuronal degeneration after various brain injuries [53]. After CCI, the density of NeuN-expressing neurons was significantly higher in N*casp8* ^−/−^ compared to control mice, both in impact lesions (Figure 5J; *p = 0.02; **p = 0.01; ***p = 0.0004) and ipsilateral semihemispheres (Figure 6B–E), approaching the values observed in brains from naïve and sham mice at 21 day (Figure 5K). Comparison with the corresponding contralateral cerebral regions revealed less pronounced declines in NeuN immunoreactivity in brains from the *casp8* knockout (Figure 5M) compared to control (Figure 5L) animals. Also, a return of NeuN immunostaining with comparable levels of this protein in the ipsi- and contralateral N*casp8* ^−/−^ regions was noted at 21 day following brain trauma (Figure 5M, N). The differences we observed in NeuN immunoreactivity may be indicative of a protective effect of neuronal caspase 8 deletion on survival of neurons, but it should be noted that loss of NeuN immunostaining theoretically could be attributed to depletion of NeuN protein and not necessarily to neuronal disintegration or death [53], [54].

Microtubule-associated proteins (MAPs), such as MAP2 and tau, are known to be sensitive markers of neuronal injury. In our TBI study, loss of MAP2 immunostaining following CCI indicated an early vulnerability of the microtubular cytoskeleton to brain trauma not only in the contused but also to a lesser extent in the contralateral cortex (Figure 6F1–F4). A correlation was observed between loss of MAP2 protein and neuronal damage as revealed by Masson's trichrome staining. Comparisons of caspase 8 deficient and control mice revealed that average levels of MAP2 protein remained higher in the impact lesions of N*casp8* ^−/−^ mice compared to control animals (*p = 0.002 at 6 h) (Figure 5O, P). We also analyzed phospho-tau levels by IHC. Hyperphosphorylation impairs the microtubule binding function of tau, ultimately leading to the degeneration of the affected neurons in a range of neuronal insults [55]. At 21 days after CCI, levels of hyperphosphorylated forms of tau protein in the ipsilateral semihemispheres of the brain-injured control mice exceeded by at least two-fold the phospho-tau protein levels in mice with neuronal deletion of caspase 8 (*p = 0.001) (Figures 5Q and 6G–J).

### Reduced cell death and caspase processing in injured brains of mice with neuronal caspase 8 deficiency

To qualitatively and quantitatively characterize neuronal cell death, we applied measurements of colorimetric intensity to a histochemical Masson's trichrome stain [35]. Morphologic criteria identified with this stain assist in distinguishing degenerating and ischemic neurons (chromatin clumps are basophilic - “dark blue neurons”, while the rest of the cell is eosinophilic) and shrunken necrotic neuronal cells characterized by brightly eosinophilic cytoplasm [35]. The percentage of degenerating cells in lesions was significantly higher in control mice compared to N*casp8* ^−/−^ animals (*p = <.0001; **p = 0.003; ***p = 0.02) (Figure 6K1). Similar conclusions were reached when the number of degenerating cells per lesion area (n/mm^2^) was calculated (*p = 0.0001; **p<.0001; ***p = 0.05; ****p = 0.01) (Figure 6K2).

Apoptosis contributes to neuronal cell death. Caspase 3 is activated by most apoptotic stimuli, becoming proteolytically cleaved to generate immunodetectable neo-epitopes [56]. In our TBI experiments, the percentage of cells with cleaved caspase 3 immunopositivity was elevated at 2 h post CCI in the lesion and penumbra, declining at 21 day after brain trauma. Although showing a similar kinetics of caspase 3 expression, the percentage of cells with caspase 3-positive cells remained significantly lower in the lesion and penumbra region of *Ncasp8* ^−/−^ mice compared to control animals (Figure 6L1; *p = 0.02). Similar observations were made when the density of cleaved caspase 3-positive cells was calculated (*p = 0.02; **p = 0.03) (Figure 6L2).

Caspase 8 cleaves and activates the effector caspase 3 to cleave PARP thus connecting this protease to the mitochondrial pathway for cell death (reviewed in [12]). By immunoblotting, we observed a depletion of the caspase 8 proform in the impact lesion samples from the control CRE3 and *Casp8* ^fl/fl^ but not *Ncasp8* ^−/−^ mice; caspase 8 proform was detected in contralateral hemispheres (CLH) of all experimental groups (Figure 6M). Strong bands corresponding to cleaved PARP were recognized in lesion samples from the control CRE3 and *Casp8^fl/fl^* but not *Ncasp8* ^−/−^ mice. Barely detectable cleaved PARP levels were noted in the contralateral hemisphere samples (Figure 6M).

The c-Jun N-terminal protein kinase (JNK) signaling pathway modulates the activity of several Bcl-2 family proteins, promoting apoptosis [57], [58]. At 2 days following CCI, the frequency or density of cells with nuclear phospho-c-Jun in the lesion and surrounding penumbra significantly increased in control (Figure 6N–R) but not in N*casp8* ^−/−^ (Figure 6S–X) mice (Figure 6Y,Z) (Y: *p = 0.03; Z: *p = 0.02; **p = 0.05). Nuclear immunoreactivity of phospho-c-Jun was barely present in the contralateral semihemisphere of control mice, thus confirming that the elevated nuclear expression of c-Jun is trauma-related.

### Assessment of the blood-brain barrier, reactive gliosis and inflammatory response in the post-traumatic brains

**Figure 7:**
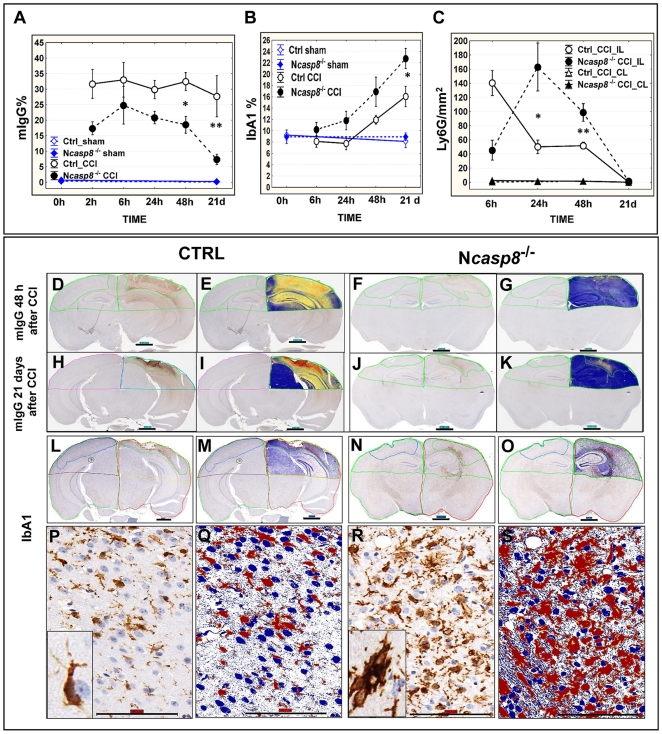
Immunohistochemical assessment of mIgG extravasation, microglia activation, and neutrophil infiltration in post-traumatic mouse brains. (**A**) Percentage of mIgG positive immunostaining was determined at various times in lesion or corresponding ipsilateral areas of the control (Ctrl) and N*casp8* ^−/−^ sham- or CCI-treated mice (8–10 mice per group) (mean±SEM; *p = 0.02; **p = 0.04). (**B**) Percentage of IbA1 immunopositive cells was compared in ipsilateral semihemispheres of the control *vs* N*casp8* ^−/−^ sham- or CCI-treated mice (*p = 0.02). (**C**) Neutrophil density (n/mm^2^) was determined by Ly6G immunostaining and compared in ipsilateral (IL) and contralateral (CL) semihemispheres of the CRE3 (Ctrl) and N*casp8* ^−/−^ mice at various times after CCI (*p = 0.03; **p = 0.02). Examples of digital (**D, F, H, J**) and mark-up (**E, G, I, K**) images of mIgG immunostaining are presented in brains from the control and N*casp8* ^−/−^ mice 48 h (**D–G**) and 3 weeks (**H–K**) after CCI. Brown color (DAB) on digital images and red, orange and yellow pixels on mark-up images denote positive immunostaining (magnification scale bar = ∼1000 µm). Digital (**L, N, P, R**) and mark-up (**M, O, Q, S**) images of the IbA1 (**L–S**) immunostainings are presented in the ipsilateral semihemispheres of the control (**L, M, P, Q**) and N*casp8* ^−/−^ (**N, O, R, S**) mice after brain trauma. Magnification scale bar = ∼100 µm. Insets show 800–1,200× magnification of activated microglia (**P, R**).

Post-traumatic alterations in the blood-brain barrier (BBB) were determined immunohistochemically by assessing extravasation of mouse immunoglobulin G (mIgG). The immunostaining demonstrated greater extravasation of mIgG in impact lesion/penumbra and ipsilateral semihemispheres of the control compared to the N*casp8* ^−/−^ mice (Figure 7A, D–K). The extravasation area decreased significantly by 21 days post CCI in the *casp8* knockout but not in the control animals (*p = 0.02; **p = 0.04) (Figure 7A).

A comparison of microglia density between the experimental groups was accomplished by measuring the percentage of IbA1 positive cells in the annotated ipsilateral semihemispheres at different time points after CCI (Figure 7B). The dynamics of changes in microglia prevalence were similar in both experimental groups with higher density of activated microglia with hypertrophic soma and thick processes in the N*casp8* ^−/−^ mice, most pronounced at 21 day post CCI (Figure 7B (*p = 0.02) and L–S). TBI causes active astrogliosis, which may cause glial scar formation and promote activation of microglia [59]. Increased frequencies of GFAP-immunopositive activated astroglia were observed in ipsilateral regions of both control and N*casp8* ^−/−^) mice compared to the contralateral semihemispheres, with no statistically significant difference between the control and N*casp8* ^−/−^ mice (not shown). Recent studies revealed that reactive astrocytes undergo substantial molecular changes that dictate their beneficial function for neuronal survival [60]. Histological examination of the leukocyte infiltration into brain hemispheres following CCI indicated a delayed influx of neutrophils (Ly6G+ cells) into the damaged area in mice lacking neuronal caspase 8 compared to controls (Figure 7C; *p = 0.03; **p = 0.02).

### Caspase 8 deletion protects against seizure-induced brain injury caused by kainic acid (KA)

**Figure 8:**
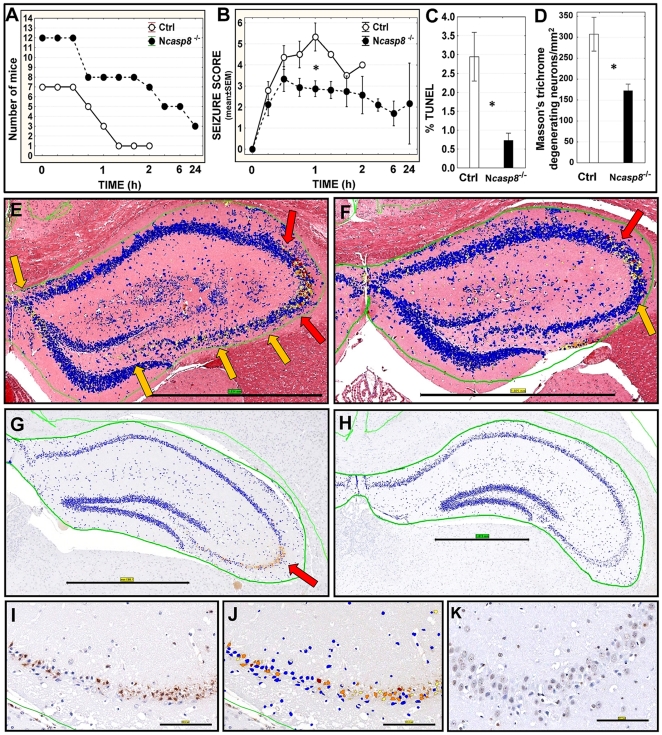
Caspase 8 deficiency protects neurons in in vivo KA-induced seizure mouse model. (**A**) Mouse survival was monitored for 24 hours after exposure of control (n = 8) and N*casp8* ^−/−^ mice (n = 11) to KA. Non-linear x-axis corresponds to time following KA injection. (**B**) Seizure scores for control and N*casp8* ^−/−^ mice were determined every 15 min for 2 h and at longer intervals thereafter [40] [41]. Percentage of TUNEL-positive neurons (**C**) and density of degenerating neurons (Masson's trichrome stain) (**D**) were assessed in the hippocampi of the control and N*casp8* ^−/−^ mice (mean±SEM). *P* values result from Student's t-test (**B**: *p = 0.008; **C**: *p = 0.002; **D**: *p = 0.005). Colorimetric intensity measurements were applied to Masson's trichrome-stained control (**E**) and N*casp8* ^−/−^ (**F**) hippocampi to obtain density of degenerating neurons in the annotated area (mark-up images). Arrows indicate hippocampal CA3–CA4 sectors and the hilus (**E**) and the CA3 sector (**F**). Apoptotic neurons were visualized by TUNEL assay (**G, H**), and cleaved-caspase 3 immunostaining (**I–K**) as presented on virtual (**I, K**) and mark-up (**G, H, J**) images of the hippocampal regions of the control (**G, I, J**) and N*casp8* ^−/−^ (**H, K**) mice. Brown color (DAB) on digital images and red, orange and yellow pixels on mark-up images denote positive staining. The arrow indicates TUNEL positive neurons in the CA3–CA4 sector.

Systemic administration of excitotoxic glutamate receptor agonist KA induces seizures and subsequent neuronal damage [61], [62]. We treated N*casp8* ^−/−^ and control mice with KA to investigate the role of caspase 8 in excitotoxicity-induced brain injury. Observation and scoring of convulsions revealed shorter mouse survival (Figure 8A) and greater severity of seizures (Figure 8B; *p = 0.008) in the control group compared to the N*casp8* ^−/−^ mice. To determine whether neuronal ablation of caspase 8 plays a protective role in KA-induced hippocampal cell death, the numbers of apoptotic and degenerating neurons were assessed by TUNEL assay (Figure 8C (*p = 0.002), G, H) and Masson's trichrome stain (Figure 8D (*p = 0.005), E, F) in hippocampal regions. Neuronal cell death (particularly abundant in the CA3 region) was decreased by caspase 8 deletion, as assessed by both methods. Results of cleaved-caspase-3 immunostaining confirmed this observation (Figure 8I–K).

## Discussion

Acute brain injury constitutes a leading cause of disability and death. Devising therapeutic strategies for ameliorating irreversible loss of neurological function from these disorders begins with identifying targets for drug discovery. In this study, we provide evidence that neuron-specific deletion of caspase 8 reduces brain damage and improves functional outcomes induced by CCI in mice, suggesting an important role for this caspase in the pathophysiology of brain trauma. The protective effect of neuronal ablation of caspase 8 was evident by reduced lesion and cavity volumes, the lower neuropathological scores, reduced frequencies of neuronal cell death, reduced numbers of cells with cleaved caspase 3, better learning performance, superior retention of spatial memory and improved sensorimotor function. Interestingly, better learning performance and superior memory retention were also observed in aged N*casp8* ^−/−^ mice with no brain injury. Alterations in CNS are known to underlie age-related impairment of motoric function. In the wire grip test, age-related decline in motoric performance is reduced in the *casp8* knockout mice compared to the age-matched controls. Further studies will be necessary to examine the effects of neuronal depletion of caspase 8 on learning and motoric function and to provide clues as to the mechanisms by which neuronal deletion of caspase 8 affects cognitive and behavioral function.

Neuronal caspase 8 deficiency also protected against neuronal injury caused by KA-induced excitotoxicity and seizures. Excitotoxicity contributes to secondary damage and cell death following acute brain injury [63] and is caused by excessive release of excitatory amino acids such as glutamate and aspartate that stimulate various glutamate receptors [64]. Neuronal cell death in hippocampus was diminished by caspase 8 deletion.

N*casp8* ^−/−^ mice showed reduced phospho-c-Jun levels after TBI. Inhibition of c-Jun activity through the expression of a dominant negative c-Jun mutant or injection of neutralizing antibodies was shown to protect neurons from apoptosis induced by withdrawal of nerve growth factor (NGF) or chronic depolarization [65], [66], [67]. Thus, the improved outcomes in N*casp8* ^−/−^ mice may be, at least in part, attributable to reduced signaling through pathways leading to c-Jun phosphorylation. In this regard, caspases have been reported to cleave and activate kinases upstream of JNK [68], which could explain why caspase 8 deficiency reduces c-Jun phosphorylation. Our finding that c-Jun phosphorylation occurs downstream of caspase 8 in *in vivo* models of acute brain injury provides a novel mechanistic insight and argues against using chemical inhibitors of c-Jun kinases (JNKs) as a therapeutic strategy for amelioration of neurotrauma, favoring targeting of caspase 8.

Diffuse axonal injury is a primary feature of head trauma and one of the most frequent causes of morbidity [69]. In humans, a post-translationally modified form of tau in the cerebrospinal fluid (CSF) of TBI patients was elevated over 1000-fold when compared to non-TBI patients, and assisted in quantification of axonal damage, with decreased levels of this protein associated with clinical improvement [69]. Thus, reduced phospho-tau protein levels in the injured semihemispheres of *casp8* knockout mice at 21 days post-TBI may be predictive of attenuated axonal injury responses. This novel observation warrants further investigation and indicates a potential for utilizing our mouse line for future exploration of TBI, Alzheimer's disease (AD), and a possible link between both pathologies. In this regard, it may be relevant that a history of TBI is one of the strongest risk factors identified to date for risk of AD (reviewed in [70]).

Loss of MAP2 immunostaining suggests an early vulnerability of the microtubular cytoskeleton to brain trauma. However, the observed subsequent increase in MAP2 immunoreactivity in N*casp8* ^−/−^ brains provides evidence that this alteration was more commonly reversible in caspase 8-deprived neurons, consistent with improved overall neuronal survival.

Enhanced microgliosis was observed in N*casp8* ^−/−^ brains, indicating that microglia activation does not require a contribution from caspase 8 expressing neurons. A primarily neuroprotective role of activated microglia after ischemia has been proposed, rendering anti-inflammatory treatment within the protective time window of microglia counterintuitive [71]. Microglia are noted for their production of cytokines, including TNFα. In the absence of caspase 8, TNFα exerts a primarily cytoprotective phenotype mediated largely by NF-κB (reviewed in [72]). In addition, our animal model results demonstrate that caspase 8 modulates the kinetics of neutrophil infiltration into damaged brain, suggesting an impact on generation of chemotactic regulators.

Growing evidence suggests that the extrinsic apoptotic pathway contributes to neuronal cell death in TBI. Elevated expression of the TNF family death receptor Fas and its ligand FasL in neurons was observed after CCI injury in rats [73]. Previously, we observed caspase 8 activation following CCI in adult rats [42]. Proteolysis of caspase 8 has been reported in rat brains after fluid percussion injury [74], and in human brains after head trauma [75]. Inhibiting caspase 8 by peptidyl caspase 8 inhibitor isoleucinyl-glutamyl-threoninyl-aspartyl-aldehyde (IETD-CHO) reportedly reduces ischemic brain injury in newborn rats [76]. While providing further evidence for a role of caspase 8 in neuronal cell death, such peptidyl protease inhibitors are known to cross-react on other members of the caspase family, limiting interpretations [77]. Additionally, upstream activators of caspase 8, such as Fas-associated DISC formation, have been reported in neurons after TBI in mice and humans [78]. Fas inhibition in *Fas^lpr/lpr^* mice reduced cell death, and improved function in rodent spinal cord injury [79]. TBI experiments performed on TNFα-deficient mice revealed an early but not sustained neuroprotective effect of TNFα inhibition [80]. However, dual deficiency of TNFα and Fas in mice subjected to CCI significantly reduced long-term brain tissue damage in both immature and adult mice, suggesting that protective effects of this double gene deletion are age-independent and long-lasting [81]. In our study, *casp8*-ablated cultured neurons show significant resistance to TNF-α in both immature and mature neurons, whereas only partial protection against staurosporine-induced cell death was observed for the mature but not for the immature neuronal cells, corroborating observations from *casp8* gene knockout experiments that caspase 8 activation is not necessary for caspase 9-mediated apoptosis [18].

While ablating caspase 8 expression would be expected to inhibit apoptosis induced by TNF family death receptors, some TNF-family cytokine receptors reportedly induce non-apoptotic cell death, raising concerns about whether interfering with caspases would be sufficient to provide neuroprotection *in vivo*. In particular, TNFR1 can stimulate the activation of a necrotic cell death pathway involving kinases Rip1 and Rip3 [82], in addition to apoptotic pathways mediated by caspase 8. Additional concerns about the effects of caspase inhibition arise from reports that caspases antagonize autophagic cell death mechanisms [12]. Induction of Rip1-dependent autophagy (instead of necrosis) was reported after exposure to zVAD-fmk or specific knockdown of caspase 8 using RNA interference (RNAi) in L929 cells [83], [84], suggesting that the absence of caspase activity favors induction of autophagic cell death. Also lymphocytes in which caspase 8 was reduced by RNAi showed an increased tendency towards autophagy [83]. While associated with cell death, autophagy is often viewed as a survival mechanism during times of nutrient insufficiency and hypoxia (ATP depletion) (reviewed in [85]). With respect to brain injury, autophagy was shown to be persistently activated after TBI, and was proposed to represent a protective mechanism [86]. Thus, if caspases oppose autophagy as previously suggested [87], then gene ablation of caspase 8 would be anticipated perhaps to promote autophagy, which might enhance neuronal survival in the setting of decreased ATP resulting from acute brain injury caused by TBI.

Following TBI and KA-induced seizures, we observed reduced apoptosis of neurons in brains of mice lacking neuronal caspase 8, as demonstrated by Masson's trichrome and TUNEL stainings or cleaved caspase 3 immunostainings. Reduced lesion cavity volume in N*casp8* ^−/−^ mice subjected to CCI, as well as improved neuropathological scores, indicated that necrotic processes are less (not more) pronounced in brains from caspase 8 knockout mice compared to control animals, thus alleviating concerns about whether interfering with caspase 8 would promote increased brain damage by necrosis.

It is noteworthy that hundreds of measurements conducted in this study were done using image analysis algorithms. Unlike semiquantitative manual scoring, computer-based image analysis permits objective quantification on entire tissue sections or anatomical regions of interest.

In addition to *in vivo* models of brain injury, we have utilized *in vitro* models including primary cortical neuron cultures and organotypic brain slices to characterize effects of conditional deletion of *caspase 8* on neuronal survival and death. Together, these results provide additional evidence of protective effect of neuronal deletion of caspase 8, corroborating the *in vivo* data. For undertaking these studies, we established an improved organotypic whole brain slice culture method using tissue from adult (4–8 month old) mice, wherein viable brain structures are maintained for up to 14 days, as evidenced by preserved morphological architecture, expression of neuronal markers (NeuN, β3-tubulin), and concomitant glial reactions. Gradually declining levels of MAP2 protein in brain slices indicate a time-dependent increase in neuronal vulnerability in these brain slice cultures and may be used to monitor viability of neurons in electrophysiological studies. Application of a polarity sensitive indicator for viability and apoptotis (pSIVA) facilitated characterization of neuronal cell death and survival in 14-day-old PNC. Thus, we developed a method of long-term whole brain slice cultures (BOSCS) using brain tissue derived from adult mice – a technique that has rarely been accomplished [88]. This model seems to be particularly advantageous in studies of genetically engineered mice.

In summary, the present findings establish a functionally important role *in vivo* for caspase 8 in neuronal cell death following acute brain injury, and encourage the development of therapeutics that target this protease as potential countermeasures for TBI and perhaps other causes of acute brain injury. It should be noted however that humans contain an additional caspase not produced in mice, namely caspase 10, that may be functionally redundant with caspase 8. Therefore, therapeutic strategies should also consider caspase 10, which is structurally and functionally highly similar to caspase 8 (reviewed in [89]), and thus likely to be sensitive to the same interventions that neutralize caspase 8.

## References

[1] Bredesen DE, Rao RV, Mehlen P (2006). Cell death in the nervous system.. Nature.

[2] Chowdhury I, Tharakan B, Bhat GK (2008). Caspases - an update.. Comp Biochem Physiol B Biochem Mol Biol.

[3] Ashkenazi A, Dixit VM (1998). Death receptors: signaling and modulation.. Science.

[4] Micheau O, Tschopp J (2003). Induction of TNF receptor I-Mediated Apoptosis via Two Sequential Signaling Complexes.. Cell.

[5] Scaffidi C, Fulda S, Srinivasan A, Friesen C, Li F (1998). Two CD95 (APO-1/Fas) signaling pathways.. EMBO J.

[6] Zou H, Li Y, Liu X, Wang X (1999). An APAF-1 cytochrome c multimeric complex is a functional apoptosome that activates procaspase-9.. J Biol Chem.

[7] Stennicke HR, Jürgensmeier JM, Shin H, Deveraux Q, Wolf BB (1998). Pro-caspase-3 is a major physiologic target of caspase-8.. J Biol Chem.

[8] Ng FWH, Nguyen M, Kwan T, Branton PE, Nicholson DW (1997). p28 Bap31, a Bcl-2/Bcl-X_L_-and procaspase-8-associated protein in the endoplasmic reticulum.. J Cell Biol.

[9] Gervais FG, Singaraja R, Xanthoudakis S, Gutekunst CA, Leavitt BR (2002). Recruitment and activation of caspase-8 by the Huntingtin-interacting protein Hip-1 and a novel partner Hippi.. Nat Cell Biol.

[10] Zhang H, Xu Q, Krajewski S, Krajewska M, Xie Z (2000). BAR: An apoptosis regulator at the intersection of caspase and bcl-2 family proteins.. Proc Natl Acad Sci USA.

[11] Roth W, Kermer P, Krajewska M, Welsh K, Davis S (2003). Bifunctional apoptosis inhibitor (BAR) protects neurons from diverse cell death pathways.. Cell Death & Differ.

[12] Vandenabeele P, Vanden Berghe T, Festjens N (2006). Caspase inhibitors promote alternative cell death pathways.. Sci STKE.

[13] Hou W, Han J, Lu C, Goldstein LA, Rabinowich H (2010). Autophagic degradation of active caspase-8: A crosstalk mechanism between autophagy and apoptosis.. Autophagy.

[14] Velier JJ (1999). Caspase-8 and caspase-3 are expressed by different populations of cortical neurons undergoing delayed cell death after focal stroke in the rat.. J Neurosci.

[15] Harrison DC, Davis RP, Bond BC, Campbell CA, James MF (2001). Caspase mRNA expression in a rat model of focal cerebral ischemia.. Brain Res Mol Brain Res.

[16] Henshall DC (2001). Cleavage of bid may amplify caspase-8-induced neuronal death following focally evoked limbic seizures.. Proc Natl Acad Sci USA.

[17] Li T, Lu C, Xia Z, Xiao B, Luo Y (2006). Inhibition of caspase-8 attenuates neuronal death induced by limbic seizures in a cytochrome c-dependent and Smac/DIABLO-independent way.. Brain Res.

[18] Varfolomeev E, Schuchmann M, Luria V, Chiannilkulchai N, Beckmann J (1998). Targeted disruption of the mouse caspase 8 gene ablates cell death inducation by the TNF receptors, Fas/Apo1, and DR3 and is lethal prenatlally.. Immunity.

[19] Taoufik E, Valable S, Muller GJ, Roberts ML, Divoux D (2007). FLIP(L) protects neurons against in vivo ischemia and in vitro glucose deprivation-induced cell death.. J Neurosci.

[20] Bagnoli M, Canevari S, Mezzanzanica D (2010). Cellular FLICE-inhibitory protein (c-FLIP) signalling: a key regulator of receptor-mediated apoptosis in physiologic context and in cancer.. Int J Biochem Cell Biol.

[21] Banares S, Zeh K, Krajewska M, Kermer P, Baribault H (2005). Novel pan-neuronal Cre-transgenic line for conditional ablation of genes in the nervous system.. Genesis.

[22] Krajewska M, Banares S, Zhang EE, Huang X, Scadeng M (2008). Development of diabesity in mice with neuronal deletion of Shp2 tyrosine phosphatase.. Am J Pathol.

[23] Salmena L, Lemmers B, Hakem A, Matysiak-Zablocki E, Murakami K (2003). Essential role for caspase 8 in T-cell homeostasis and T-cell-mediated immunity.. Genes Dev.

[24] Li SH, Li H, Torre ER, Li XJ (2000). Expression of huntingtin-associated protein-1 in neuronal cells implicates a role in neuritic growth.. Mol Cell Neurosci.

[25] Ahlemeyer B, Baumgart-Vogt E (2005). Optimized protocols for the simultaneous preparation of primary neuronal cultures of the neocortex, hippocampus and cerebellum from individual newborn (P0.5) C57Bl/6J mice.. J Neurosci Methods.

[26] Liu D, Gharavi R, Pitta M, Gleichmann M, Mattson MP (2009). Nicotinamide prevents NAD+ depletion and protects neurons against excitotoxicity and cerebral ischemia: NAD+ consumption by SIRT1 may endanger energetically compromised neurons.. Neuromolecular Med.

[27] Kim YE, Chen J, Langen R, Chan JR (2010). Monitoring apoptosis and neuronal degeneration by real-time detection of phosphatidylserine externalization using a polarity-sensitive indicator of viability and apoptosis.. Nat Protoc.

[28] Stoppini L, Buchs PA, Muller D (1991). A simple method for organotypic cultures of nervous tissue.. J Neurosci Methods.

[29] You Z, Savitz SI, Yang J, Degterev A, Yuan J (2008). Necrostatin-1 reduces histopathology and improves functional outcome after controlled cortical impact in mice.. J Cereb Blood Flow Metab.

[30] Fox GB, Faden AI (1998). Traumatic brain injury causes delayed motor and cognitive impairment in a mutant mouse strain known to exhibit delayed Wallerian degeneration.. J Neurosci Res.

[31] Bermpohl D, You Z, Korsmeyer SJ, Moskowitz MA, Whalen MJ (2006). Traumatic brain injury in mice deficient in Bid: effects on histopathology and functional outcome.. J Cereb Blood Flow Metab.

[32] Sinz EH, Kochanek PM, Dixon CE, Clark RS, Carcillo JA (1999). Inducible nitric oxide synthase is an endogenous neuroprotectant after traumatic brain injury in rats and mice.. J Clin Invest.

[33] Hall ED, Yonkers PA, McCall JM, Braughler JM (1988). Effects of the 21-aminosteroid U74006F on experimental head injury in mice.. J Neurosurg.

[34] Fujimoto ST, Longhi L, Saatman KE, Conte V, Stocchetti N (2004). Motor and cognitive function evaluation following experimental traumatic brain injury.. Neurosci Biobehav Rev.

[35] Krajewska M, Smith LH, Rong J, Huang X, Hyer ML (2009). Image analysis algorithms for immunohistochemical assessment of cell death events and fibrosis in tissue sections.. J Histochem Cytochem.

[36] Paxinos GaF, K.B.J. (2001). The mouse brain in stereotaxic coordinates.

[37] Rosen GD, Harry JD (1990). Brain volume estimation from serial section measurements: a comparison of methodologies.. J Neurosci Methods.

[38] Ruifrok AC, Johnston DA (2001). Quantification of histochemical staining by color deconvolution.. Anal Quant Cytol Histol.

[39] Krajewski S, Huang X, Krajewska M (2009). Multiple antigen detection (MAD) western blotting.. Methods Mol Biol.

[40] Yang DD, Kuan CY, Whitmarsh AJ, Rincon M, Zheng TS (1997). Absence of excitotoxicity-induced apoptosis in the hippocampus of mice lacking the Jnk3 gene.. Nature.

[41] Zhang J (2001). Dna fragmentation factor 45 mutant mice exhibit resistance to kainic acid-induced neuronal cell death.. Biochem Biophys Res Commun.

[42] Beer R, Franz G, Krajewski S, Pike BR, Hayes RL (2001). Temporal and spatial profile of caspase-8 expression and proteolysis after experimental traumatic brain injury.. J Neurochem.

[43] Cho S, Wood A, Bowlby MR (2007). Brain slices as models for neurodegenerative disease and screening platforms to identify novel therapeutics.. Curr Neuropharmacol.

[44] Yoon JJ, Nicholson LF, Feng SX, Vis JC, Green CR (2010). A novel method of organotypic brain slice culture using connexin-specific antisense oligodeoxynucleotides to improve neuronal survival.. Brain Res.

[45] Finley M, Fairman D, Liu D, Li P, Wood A (2004). Functional validation of adult hippocampal organotypic cultures as an in vitro model of brain injury.. Brain Res.

[46] Xiang Z, Hrabetova S, Moskowitz SI, Casaccia-Bonnefil P, Young SR (2000). Long-term maintenance of mature hippocampal slices in vitro.. J Neurosci Methods.

[47] Matus A (1988). Microtubule-associated proteins: their potential role in determining neuronal morphology.. Annu Rev Neurosci.

[48] Romito-DiGiacomo RR, Menegay H, Cicero SA, Herrup K (2007). Effects of Alzheimer's disease on different cortical layers: the role of intrinsic differences in Abeta susceptibility.. J Neurosci.

[49] Sprick MR, Weigand MA, Rieser E, Rauch CT, Juo P (2000). FADD/MORT1 and caspase-8 are recruited to TRAIL receptors 1 and 2 and are essential for apoptosis mediated by TRAIL receptor 2.. Immunity.

[50] Falschlehner C, Emmerich CH, Gerlach B, Walczak H (2007). TRAIL signalling: decisions between life and death.. Int J Biochem Cell Biol.

[51] Igarashi T, Huang TT, Noble LJ (2001). Regional vulnerability after traumatic brain injury: gender differences in mice that overexpress human copper, zinc superoxide dismutase.. Exp Neurol.

[52] Sugawara T, Lewen A, Noshita N, Gasche Y, Chan PH (2002). Effects of global ischemia duration on neuronal, astroglial, oligodendroglial, and microglial reactions in the vulnerable hippocampal CA1 subregion in rats.. J Neurotrauma.

[53] Collombet JM, Masqueliez C, Four E, Burckhart MF, Bernabe D (2006). Early reduction of NeuN antigenicity induced by soman poisoning in mice can be used to predict delayed neuronal degeneration in the hippocampus.. Neurosci Lett.

[54] Unal-Cevik I, Kilinc M, Gursoy-Ozdemir Y, Gurer G, Dalkara T (2004). Loss of NeuN immunoreactivity after cerebral ischemia does not indicate neuronal cell loss: a cautionary note.. Brain Res.

[55] Hanger DP, Anderton BH, Noble W (2009). Tau phosphorylation: the therapeutic challenge for neurodegenerative disease.. Trends Mol Med.

[56] Kumar S (1997). The apoptotic cysteine protease CPP32.. Int J Biochem Cell Biol.

[57] Okuno S, Saito A, Hayashi T, Chan PH (2004). The c-Jun N-terminal protein kinase signaling pathway mediates Bax activation and subsequent neuronal apoptosis through interaction with Bim after transient focal cerebral ischemia.. J Neurosci.

[58] Love S (2003). Apoptosis and brain ischaemia.. Prog Neuropsychopharmacol Biol Psychiatry.

[59] Stoica B, Byrnes K, Faden AI (2009). Multifunctional drug treatment in neurotrauma.. Neurotherapeutics.

[60] Escartin C, Bonvento G (2008). Targeted activation of astrocytes: a potential neuroprotective strategy.. Mol Neurobiol.

[61] Ferkany JW, Zaczek R, Coyle JT (1984). The mechanism of kainic acid neurotoxicity.. Nature.

[62] Fujikawa DG (2005). Prolonged seizures and cellular injury: understanding the connection.. Epilepsy Behav.

[63] Vink R, Nimmo AJ (2009). Multifunctional drugs for head injury.. Neurotherapeutics.

[64] Olney JW, Labruyere J, Price MT (1989). Pathological changes induced in cerebrocortical neurons by phencyclidine and related drugs.. Science.

[65] Estus S, Zaks WJ, Freeman RS, Gruda M, Bravo R (1994). Altered gene expression in neurons during programmed cell death: identification of c-jun as necessary for neuronal apoptosis.. J Cell Biol.

[66] Ham J, Babij C, Whitfield J, Pfarr CM, Lallemand D (1995). A c-Jun dominant negative mutant protects sympathetic neurons against programmed cell death.. Neuron.

[67] Whitfield J, Neame SJ, Paquet L, Bernard O, Ham J (2001). Dominant-negative c-Jun promotes neuronal survival by reducing BIM expression and inhibiting mitochondrial cytochrome c release.. Neuron.

[68] Liu J, Lin A (2005). Role of JNK activation in apoptosis: a double-edged sword.. Cell Res.

[69] Zemlan FP, Rosenberg WS, Luebbe PA, Campbell TA, Dean GE (1999). Quantification of axonal damage in traumatic brain injury: affinity purification and characterization of cerebrospinal fluid tau proteins.. J Neurochem.

[70] Johnson VE, Stewart W, Smith DH (2010). Traumatic brain injury and amyloid-beta pathology: a link to Alzheimer's disease?. Nat Rev Neurosci.

[71] Neumann J, Gunzer M, Gutzeit HO, Ullrich O, Reymann KG (2006). Microglia provide neuroprotection after ischemia.. FASEB J.

[72] Wullaert A, Heyninck K, Beyaert R (2006). Mechanisms of crosstalk between TNF-induced NF-kappaB and JNK activation in hepatocytes.. Biochem Pharmacol.

[73] Beer R, Franz G, Schopf M, Reindl M, Zelger B (2000). Expression of Fas and Fas ligand after experimental traumatic brain injury in the rat.. J Cereb Blood Flow Metab.

[74] Keane RW, Kraydieh S, Lotocki G, Alonso OF, Aldana P (2001). Apoptotic and antiapoptotic mechanisms after traumatic brain injury.. J Cereb Blood Flow Metab.

[75] Zhang X, Graham SH, Kochanek PM, Marion DW, Nathaniel PD (2003). Caspase-8 expression and proteolysis in human brain after severe head injury.. Faseb J.

[76] Feng Y, Fratkin JD, LeBlanc MH (2003). Inhibiting caspase-8 after injury reduces hypoxic-ischemic brain injury in the newborn rat.. Eur J Pharmacol.

[77] Turk B, Turk D, Salvesen GS (2002). Regulating cysteine protease activity: essential role of protease inhibitors as guardians and regulators.. Curr Pharm Des.

[78] Qiu J, Whalen MJ, Lowenstein P, Fiskum G, Fahy B (2002). Upregulation of the Fas receptor death-inducing signaling complex after traumatic brain injury in mice and humans.. J Neurosci.

[79] Casha S, Yu WR, Fehlings MG (2005). FAS deficiency reduces apoptosis, spares axons and improves function after spinal cord injury.. Exp Neurol.

[80] Scherbel U, Raghupathi R, Nakamura M, Saatman KE, Trojanowski JQ (1999). Differential acute and chronic responses of tumor necrosis factor-deficient mice to experimental brain injury.. Proc Natl Acad Sci U S A.

[81] Bermpohl D, You Z, Lo EH, Kim HH, Whalen MJ (2007). TNF alpha and Fas mediate tissue damage and functional outcome after traumatic brain injury in mice.. J Cereb Blood Flow Metab.

[82] Vandenabeele P, Declercq W, Van Herreweghe F, Vanden Berghe T (2010). The role of the kinases RIP1 and RIP3 in TNF-induced necrosis.. Sci Signal.

[83] Yu L, Alva A, Su H, Dutt P, Freundt E (2004). Regulation of an ATG7-beclin 1 program of autophagic cell death by caspase-8.. Science.

[84] Yu L, Wan F, Dutta S, Welsh S, Liu Z (2006). Autophagic programmed cell death by selective catalase degradation.. Proc Natl Acad Sci U S A.

[85] Eskelinen EL, Saftig P (2009). Autophagy: a lysosomal degradation pathway with a central role in health and disease.. Biochim Biophys Acta.

[86] Liu CL, Chen S, Dietrich D, Hu BR (2008). Changes in autophagy after traumatic brain injury.. J Cereb Blood Flow Metab.

[87] Djavaheri-Mergny M, Maiuri MC, Kroemer G (2010). Cross talk between apoptosis and autophagy by caspase-mediated cleavage of Beclin 1.. Oncogene.

[88] Staal JA, Alexander SR, Liu Y, Dickson TD, Vickers JC (2011). Characterization of Cortical Neuronal and Glial Alterations during Culture of Organotypic Whole Brain Slices from Neonatal and Mature Mice.. PLoS One.

[89] Ben Moshe T, Kang TB, Kovalenko A, Barash H, Abramovitch R (2008). Cell-autonomous and non-cell-autonomous functions of caspase-8.. Cytokine Growth Factor Rev.

